# Real-Time UV Measurement With a Sun Protection System for Warning Young Adults About Sunburn: Prospective Cohort Study

**DOI:** 10.2196/25895

**Published:** 2021-05-06

**Authors:** June K Robinson, Shiv Patel, Seung Yun Heo, Elizabeth Gray, Jaeman Lim, Kyeongha Kwon, Zach Christiansen, Jeffrey Model, Jacob Trueb, Anthony Banks, Mary Kwasny, John A Rogers

**Affiliations:** 1 Northwestern University Feinberg School of Medicine Chicago, IL United States; 2 Department of Biomedical Engineering Center for Bio-Integrated Electronics, Simpson Querrey Institute for BioNanotechnology Northwestern University Evanston, IL United States; 3 Department of Preventive Medicine Northwestern University Feinberg School of Medicine Chicago, IL United States; 4 Wearifi Inc Evanston, IL United States; 5 School of Electrical Engineering Korea Advanced Institute of Science and Technology Daejeon Republic of Korea

**Keywords:** sun protection, UV dosimeter, health promotion technology, melanoma, sunburn, preventive medicine, mobile phone

## Abstract

**Background:**

Melanoma is attributable to predisposing phenotypical factors, such as skin that easily sunburns and unprotected exposure to carcinogenic UV radiation. Reducing the proportion of young adults who get sunburned may reduce the incidence of melanoma, a deadly form of skin cancer. Advances in technology have enabled the delivery of real-time UV light exposure and content-relevant health interventions.

**Objective:**

This study aims to examine the feasibility of young adults performing the following tasks daily: wearing a UV dosimeter, receiving text messages and real-time UV-B doses on their smartphone, and responding to daily web-based surveys about sunburn and sun protection.

**Methods:**

Young adults aged 18-39 years (n=42) were recruited in the United States in June 2020 via social media. Participants received the UV Guard sun protection system, which consisted of a UV dosimeter and a smartphone app. During 3 consecutive periods, intervention intensity increased as follows: real-time UV-B dose; UV-B dose and daily behavioral facilitation text messages; and UV-B dose, goal setting, and daily text messages to support self-efficacy and self-regulation. Data were self-reported through daily web-based surveys for 28 days, and UV-B doses were transmitted to cloud-based storage.

**Results:**

Patients’ median age was 22 years (IQR 20, 29), and all patients had sun-sensitive skin. Sunburns were experienced during the study by fewer subjects (n=18) than those in the preceding 28 days (n=30). In July and August, the face was the most commonly sunburned area among 13 body locations; 52% (22/42) of sunburns occurred before the study and 45% (19/42) occurred during the study. The mean daily UV-B dose decreased during the 3 periods; however, this was not statistically significant. Young adults were most often exercising outdoors from 2 to 6 PM, walking from 10 AM to 6 PM, and relaxing from noon to 2 PM. Sunburn was most often experienced during exercise (odds ratio [OR] 5.65, 95% CI 1.60-6.10) and relaxation (OR 3.69, 95% CI 1.03-4.67) relative to those that did not exercise or relax in each category. The self-reported exit survey indicated that participants felt that they spent less time outdoors this summer compared to the last summer because of the COVID-19 pandemic and work. In addition, 38% (16/42) of the participants changed their use of sun protection based on their app-reported UV exposure, and 48% (20/42) shifted the time they went outside to periods with less-intense UV exposure. A total of 79% (33/42) of the participants were willing to continue using the UV Guard system outside of a research setting.

**Conclusions:**

In this proof-of-concept research, young adults demonstrated that they used the UV Guard system; however, optimization was needed. Although some sun protection behaviors changed, sunburn was not prevented in all participants, especially during outdoor exercise.

**Trial Registration:**

ClinicalTrials.gov NCT03344796; http://clinicaltrials.gov/ct2/show/NCT03344796

## Introduction

### Background

Young adults aged between 18 and 39 years often engage in activities that expose them to high amounts of carcinogenic UV radiation [1]. Melanoma, the second most diagnosed cancer in young adults [2], is attributable to both predisposing genetic and phenotypical factors, such as having skin that easily gets sunburned, as well as unprotected exposure to UV. Leisure-time physical activity, such as sports engaged in by young adults to maintain fitness and socialize, has been found to be associated with an increased risk of melanoma [3]. Outdoor leisure-time physical activity is frequently performed with limited skin coverage by clothing and is associated with an increased risk of sunburn [4]. In contrast to the prior focus on the importance of childhood sunburns, growing epidemiological evidence indicates that sunburns sustained during all life periods, including adulthood, increase the risk of melanoma [5]. In the United States, 50.2% of young adults aged 18-29 years and 51.2% of individuals with sun-sensitive skin experience sunburn each year [6]. Thus, limiting the amount of unprotected UV exposure during young adulthood has the potential to decrease the risk of melanoma, especially among high-risk individuals. Various sun protection behaviors, such as avoiding the sun during peak UV hours (10 AM-4 PM), wearing protective clothing, and applying broad-spectrum sunscreen with a sun protection factor (SPF) of 30 or higher, are recommended.

A limited number of studies have evaluated the impact of sun protection interventions on self-reported sun protection usage by at-risk young adults. If the biological outcome of sunburn was collected in these studies, participants were asked to recall the number of sunburns in the last month, which was subject to recall bias. The use of a web-based intervention was effective in reducing UV exposure and increasing skin protection behaviors over 12 weeks [7]. In a 3-month Australian study, individuals equipped with personal UV monitors improved their sun protection behaviors on weekends compared with a control group [8]. The UV monitor provided the personal real-time UV dose; however, the cumulative UV daily dose was not determined. In addition, users sought more control over the feedback method provided by the UV monitor, which sounded an alarm at predefined UV thresholds based on anticipated sunburn associated with skin types. Specifically, participants noted that the ability to tailor the alert as a custom alarm or a subtle vibration would be more appealing. Within the same study, another group of participants received a different intervention involving sun protection notifications delivered through a mobile app. However, many users of the app reported that the notifications were repetitive and offered information that was already known.

### Objective

Findings from our past research informed the development of the sun protection mobile app, wearable UV sensor, UV Guard program, and text messages used in this study [9-11]. The wearable UV sensor collected and reported UV-B measurements in real time through an accompanying mobile app on the user’s smartphone. These UV measurements were also stored in a cloud database of personal UV exposure and were accessed daily. The purpose of this study is to evaluate the feasibility of young adult participants wearing the UV sensor each day, receiving daily sun protection messages by SMS texts, and reporting their sun exposure and protection behaviors on a web-based app daily for a span of 28 days. Sun exposure and protection and the risk of sunburn among a cohort of young adults were assessed.

## Methods

### Recruitment

In June 2020, participants were recruited by posting electronic announcements on college websites and high school alumnae organizations in the Midwestern and Southeastern United States. The announcement stated that the aim of the study was to provide real-time UV exposure to participants to prevent sunburn. Eligibility criteria were age 18-39 years, having skin that gets pink (only just perceptible reddening of the skin) after being in the sun, normally spending at least 30 consecutive minutes a day outdoors, having a home address with a direct mail address to receive the UV dosimeter, having reliable internet access, and having a smartphone with iOS version 13.0 or above that was able to support the app, be willing to wear the sensor on the wrist similar to a watch for 28 days, and complete daily web-based surveys. Young adults, who were interested in participating in the study, clicked a link provided, after which they were directed to a survey website (REDCap [Research Electronic Data Capture]) [12].

### Intervention Device

**Figure 1:**
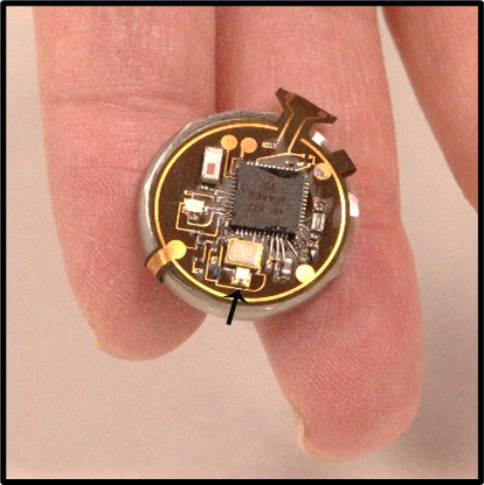
Wireless UV-B dosimeter worn on the wrist (arrow indicates a UV-B photodiode).

The directions for using the wireless miniaturized UV dosimeter with a Bluetooth connection to the smartphone and cloud-based storage of the daily UV-B dose were provided by email, and printed directions were enclosed with the device [13,14]. The device was shipped to the participants by Federal Express (Figure 1). After receiving the device, participants logged onto the web-based survey [12] to complete the REDCap survey. The Institutional Review Board of Northwestern University approved the study, and written informed consent was obtained from each participant (Multimedia Appendix 1). For people who qualified to participate in this study, an electronic gift card was sent by email at the completion of the study.

The ultra–low-power digital dosimeter platform used in this study provided continuous real-time UV-B doses wirelessly to the users’ smartphone, with immediate access to the dose received in relation to their sunburn threshold. The operation relies on a UV-B photodiode that continuously accumulates charge on a storage capacitor such that the resulting voltage corresponds directly to the exposure dose via a calibration factor. The use of this accumulation detection module with an advanced, light-adaptive electronic control circuit enabled exceptionally high levels of power efficiency for a long battery life [13]. When the exposure dose exceeded a predefined threshold value, the device automatically and wirelessly transmitted the dose value to a smartphone. For field deployment, devices such as those shown in Figure 1 were packaged into housings designed for digital watches for mounting on the wrist. This type of wearable dosimeter, with a smartphone and a cloud computing system, was used to support personalized UV-B monitoring (Figure 1).

### Study Design: Text Messages

In June and July 2019, semistructured interviews were conducted with 30 young adults (15 males and 15 females) to develop text messages using the principles of Social Cognitive Theory (self-efficacy and outcome expectancies) and qualitative data analytic methods previously reported [15,16]. As young adults wanted knowledge (sun protection tips) from an expert, a series of 15 knowledge-based messages to facilitate sun protection behavior were assessed by Likert scale (range 1-5; 1=very useful; 5=not useful) and refined to 8 items with a mean score of 1.8 (SD 0.99). Five outcome expectancy items were refined to 2 items, with a mean score of 1.6 (SD 0.75). Four self-regulation items had a mean score of 2.6 (SD 1.3). Self-efficacy items, which were not tailored to the individual behavior of users, had a mean score of 2.8 (SD 1.4).

Participants did not report if they received or read the text messages.

### Study Design: Interventional Study

**Figure 2:**
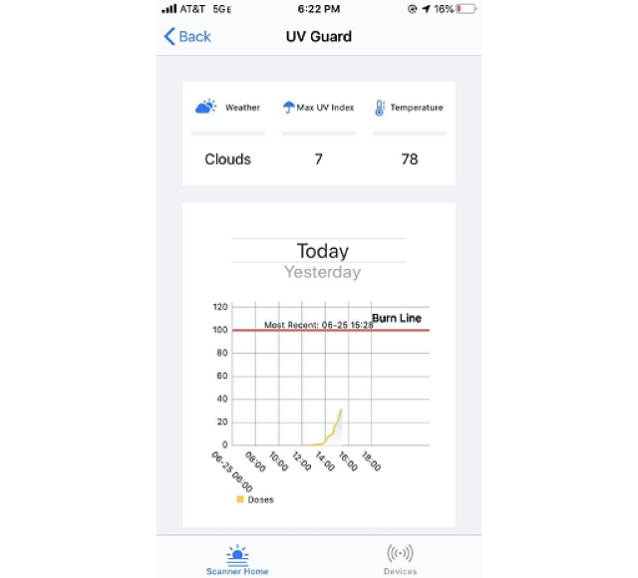
Smartphone screen showing the real-time UV-B dose in relation to the threshold dose at which the participant would get a sunburn; the predicted weather, including cloud cover; UV index; and temperature.

From July to August 2020, an interventional study with a series of components was conducted over 3 periods. Initially, participants selected their skin type based on their perceived sun sensitivity to sunburn, which corresponded to skin type I, II, or III. The minimal erythema dose (MED) for skin type I, II, and III is 200 J/m^2^, 250 J/m^2^, and 300 J/m^2^, respectively [17]. Thus, a sunburn line was established for each participant according to their self-reported skin type (Figure 2). Sunburn was defined as only just perceptible reddening of the skin (pink) at 24 hours after UV exposure [18]. Intermittent UV exposure may produce an MED equivalent to a single UV exposure, which supports the cautious approach of using cumulative UV daily exposure to warn participants of impending sunburn.

The study periods were as follows: (1) days 0-7: participants reviewed the real-time daily cumulative UV-B exposure provided on the screen of their smartphone in a graph showing the UV-B dose at which their skin would sustain a sunburn displayed on the graph with a red line (Figure 2); (2) days 8-17: UV-B dose plus daily text messages at 11 AM to enhance sun protection (Textbox 1); and (3) days 18-28: on day 18, each participant selected primary and secondary goals to improve their sun protection and indicated their intention to continue performing the sun protection. In addition to the UV-B dose, participants received text messages described in Textbox 1 whose content shifted from behavioral facilitation (knowledge) and outcome expectancy in the first 16 days to self-efficacy and self-regulation following goal setting on day 18. Young adults selected their preferred time to receive text messages.

**Daily text messages.**

**Days 0-7**

- No text message

**Day 8**

- *Remember, the purpose of sun protection is to prevent a skin cancer and keep your skin from aging. Make sure to use sun protection; sunscreen, wearing sun protective clothing, and stay in the shade when possible!* [Behavioral facilitation]

**Day 9**

- *Sun peak intensity alert! Did you know UV rays, which are the harmful rays of the sun, are strongest from 10 AM to 4 PM? Be extra careful during this time.* [Behavioral facilitation]

**Day 10**

- *Try self-tanning lotion to safely appear tanned.* [Behavioral facilitation]

**Day 11**

- *An SPF of 30 indicates protection against 97% of harmful UV rays. Apply sunscreen 15-20 min before going outside and re-apply if you’re still in the sun after two hours.* [Outcome expectancy]

**Day 12**

- *Lips need love too! Be sure to apply a lip balm, Chap Stick, and/or lipstick with an SPF of at least 30 to your lips. Lips can get a melanoma too.* [Behavioral facilitation]

**Day 13**

- *It is easy to miss spots when you are applying sunscreen. Using 2 coats helps miss fewer areas! Do one area of the body, let it dry, and then reapply another coat.* [Behavioral facilitation]

**Day 14**

- *Sunburns can happen on cloudy days. More than 80% of UV rays can pass through clouds. UV rays also reflect off water and concrete.* [Outcome expectancy]

**Day 15**

- *When shopping for a sunscreen, be sure to select one that says it is broad-spectrum, has an SPF of >30 and is water resistant. Be sure to check the expiration dates of bottles you already have.* [Behavioral facilitation]

**Day 16**

- *As you sweat, your sunscreen washes away. Be sure to reapply your sunscreen after 80 min. The new sunscreen layer is effective when the old layer is used up at 2 hours. This applies to outdoor activities like exercising, errands, and commuting!* [Behavioral facilitation]

**Day 17**

- *Keeping track of your UV exposure each day will get you a little closer to achieving your sun protection goals!* [Self-efficacy]

**Day 18**

- *Pay it forward! Text or call a family member and friend and remind them of the importance of sun protection!* [Self-efficacy]

**Day 18 Goal setting**

- *How will you meet your goal to engage in sun-protected outdoor activity? (Select your first and second choices) Tomorrow, I will:*
*Apply sunscreen to all areas of my body that may be exposed to the sun.*
*Apply sunscreen before I go outdoors.*
*Wear a hat when I am outdoors.*
*Wear a shirt that covers my shoulders when I am outdoors.*
*Plan my outdoor activities to avoid being outside from 10 AM to 2 PM.*
*Pay attention to the strength of the sun by checking the UV report 15 min after I go outside.*
*Be careful not to exceed the amount of UV my skin can tolerate.*

**Day 19 Goal intention**

- *You picked the goal to [...]: Do you intend to keep doing this?*

**Day 19**

- *As a pick-me-up, take a few minutes out of your day and go for a walk outdoors. Be sure to use sun protection and walk where there is the most shade!* [Self-regulation]

**Day 20**

- *Keep sunscreen someplace where you’ll see it, like near your toothpaste. Make it part of your morning routine.* [Behavioral facilitation]

**Day 21**

- *If you forget to apply sunscreen and do not have sun protective clothing or a hat, be sure to utilize shade!* [Self-regulation]

**Day 22**

- *Two-thirds of adults get a sunburn more than once each year. Pledge to not be one of them.* [Self-regulation]

**Day 23**

- *A sunburn does not always hurt or blister. If you press on skin that is slightly pink and the color goes away, you have a sunburn.* [Self-regulation]

**Day 24**

- *Try keeping a hat or sunscreen somewhere that’s easy to get when you are on-the-go, such as your car or backpack.* [Behavioral facilitation]

**Day 25**

- *A good way to figure out if a tree gives enough shade for complete sun protection is to look at the ground and see if spots of sunlight show on the ground.* [Self-regulation]

**Day 26**

- *Practicing safe sun habits is my lifelong habit.* [Self-efficacy]

**Day 27**

- *Getting in the habit of protecting your skin now will help you make it a habit for many years to come!* [Self-efficacy]

**Day 28**

- *Good for you! You met your goal of improving your sun protection.* [Self-efficacy]

Data were collected during July and August 2020. If a participant failed to complete the daily self-reported survey or the UV-B dose was not transferred to the cloud, the participant received an email reminder the next day.

The Institutional Review Board of Northwestern University approved the study protocol. Participants provided written consent and were offered a US $200 gift card after completing the final survey.

### Measures

Baseline self-reported responses included age, gender, race or ethnicity, skin type (sun sensitivity), family and personal history of skin cancer, sunburns, and body parts with a sunburn in the last 28 days; knowledge and attitudes about sunburn, sun exposure, and sun protection; estimated hours outside 10 AM and 4 PM on weekdays and weekend days in the past 28 days; and sun protection used in the past 28 days, including wearing sunscreen, wearing a shirt with sleeves or a T-shirt, wearing sunglasses, or staying in the shade (Likert scale: 1=never; 3=sometimes; 5=always]; Table 1). These measures were used in prior sun exposure and protection studies [9,19].

**Table 1:** Schedule of measures.

Measure		Day 0: baseline	Days 0-7: observe	Days 8-17: daily text messages	Day 18: structured goal setting	Days 18-28: daily text messages
**Self-report**						
	Demographics	✓				
	Sunburn in last 28 days	✓				
	Confidence and anxiety	✓	D^a^7	D16		D28
	Daily sunburn or sun protection		✓	✓	✓	✓
	Structured goal				✓	
	System Usability Scale					✓
	Willingness to continue use					✓
**Sensor**						
	Daily UV	✓	✓	✓	✓	✓
**Intervention**						
	Daily text messages			✓	✓	✓
	UV exposure visualization		✓	✓	✓	✓

^a^D: day.

**Figure 3:**
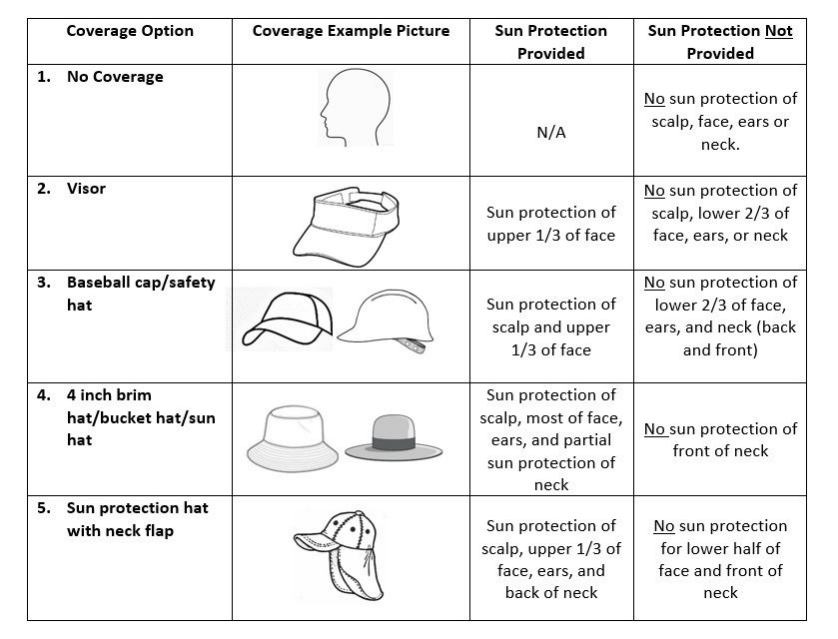
Example of a self-reported measure for clothing covering the head and the neck. N/A: not applicable.

Daily outside activities were elicited for 2-hour blocks of time (6-7:59 AM, 8-9:59 AM, 10-11:59 AM, noon-1:59 PM, 2 to 3:59 PM, and 4 to 5:59 PM). Participants also reported sun protection for the same 2-hour blocks by selecting a picture of the type of clothing worn (Figure 3) and any clothing changes during the day and reported the application of sunscreen, reapplication, and SPF of the sunscreen. Daily sunburns of 13 body parts were self-reported each evening in the web-based survey (Multimedia Appendix 2). The temperature and sun-clear or cloudy or rain-stormy conditions were obtained from the Open Weather Map for the geographic location of the participant for the 2-hour block of time used to self-report sun protection. Daily UV-B exposure for each participant was obtained from the wearable UV sensor.

UV exposure was transmitted from the personal UV dosimeter to users’ smartphone and to the cloud database. Exposure during the preceding 24 hours was downloaded each day.

On day 18, the REDCap system offered participants the opportunity to select 2 items from the following list of possible goals to implement tomorrow: (1) “Apply sunscreen to all of the areas of my body that may be exposed to the sun,” (2) “Apply sunscreen before I go outdoors,” (3) “Wear a hat when I am outdoors,” (4) “Wear a shirt that covers my shoulders when I am outdoors,” (5) “Plan my outdoor activities to avoid being outside from 10 AM to 2 PM,” (6) “Pay attention to the strength of the sun by checking the UV Guard report 15 minutes after I go outside,” and (7) “Be careful not to exceed the amount of UV my skin can tolerate.” The following day, participants were reminded of their primary goal choice and asked if they intended to keep doing it.

Anxiety was assessed with self-reported responses to 11 items ranging from 1 (strongly disagree) to 5 (strongly agree) used in previous research (range 11-55) [9]. Similarly, confidence in practicing sun protection was assessed using 11 items (range 11-55).

### Statistical Analysis

Demographic characteristics were summarized using medians and IQRs for age and counts and percentages for other demographics, including knowledge scores. Generalized linear mixed models with logit links were used to assess the estimated probabilities of probabilities of reporting daily sunburns for each day, body part, study period, and time of day, and an identity link was used to model the UV dose. Both models assumed an unstructured covariance matrix. Least square means and SDs were presented as the mean percentage of days that participants reported a sunburn within each study period after adjusting for weather (cloudy, rainy, or clear), UV dose, and the use of sun protection. In addition to type 3 tests for the main effects of the study period on reported sunburns, reported activities during the day were also examined for associations. Odds ratios (ORs) and 95% CIs are presented. Descriptive statistics were used to describe the goals and summarize the study experiences of the subjects at a study exit interview. Estimated anxiety and confidence regarding sun protection were compared across time points using repeated measures analysis of variance models and reported using means and SDs. All analyses were run using R 3.6.0 (The R Foundation) at a nominal type I error rate of 5% [20].

### Data Exclusion

One UV dosimeter malfunctioned; therefore, 1 participant’s data were excluded from the UV-B exposure reported in Table 2.

**Table 2:** Daily sunburns and UV-B dose experienced by participants and mean proportion of each day spent outside by all participants during the 3 periods (days 0-7, days 8-17, and days 18-28).

Variables			Days 0-7	Days 8-17	Days 18-28	*P* value^a^	
Population, n			42	42	42	N/A^b^	
**Individual participants**							
	**Number of sunburns, n (%)**						.55
		0	24 (57)	27 (64)	25 (59)		
		1	12 (29)	8 (19)	8 (19)		
		2	4 (10)	5 (12)	6 (14)		
		More than 2	2 (5)	2 (5)	3 (7)		
	UV-B daily dose (J/m^2^), mean (SD)^c^		91.96 (115.34)	74.97 (82.32)	62.73 (71.44)	.08	
**Sample**							
	**Proportion of each day spent outside, mean (SD)**		0.87 (0.19)	0.84 (0.21)	0.81 (0.24)	.05	
		6-7:59 AM	0.15 (0.25)	0.14 (0.20)	0.14 (0.21)	.93	
		8-9:59 AM	0.19 (0.21)	0.18 (0.20)	0.18 (0.22)	.93	
		10-11:59 AM	0.33 (0.28)	0.31 (0.23)	0.31 (0.22)	.74	
		Noon-1:59 PM	0.43 (0.29)	0.41 (0.22)	0.36 (0.24)	.20	
		2-3:59 PM	0.44 (0.26)	0.37 (0.19)	0.39 (0.25)	.24	
		4-5:59 PM	0.47 (0.27)	0.45 (0.30)	0.48 (0.28)	.67	

^a^*P* values are from the main effects of time from repeated measures general linear models (log or identity link).

^b^N/A: not applicable.

^c^The UV device malfunctioned for 1 person; thus, the participant’s data were removed.

## Results

### Participant Characteristics

A total of 44 young adults were enrolled in the study and 42 completed the 28-day study. All participants self-reported having either very sun-sensitive skin or average sun-sensitive skin (Table 3). Within 5 days of enrolling, 2 participants stopped completing the daily self-reported survey and wearing the sensor; therefore, they ceased study participation. All of the remaining 42 participants wore the UV sensor daily and completed a daily self-reported survey for 28 days. The time most frequently selected to receive text messages was 11 AM.

**Table 3:** Participant characteristics.

Characteristics		Values
Population, n		42
Age (years), median (first quartile, third quartile)		22 (20, 29)
**Gender, n (%)**		
	Female	28 (67)
	Male	14 (33)
**Race, n (%)**		
	White	36 (86)
	Asian	3 (7)
	Other	2 (5)
	Prefer not to answer	1 (2)
**Ethnicity, n (%)**		
	Non-Hispanic	39 (93)
	Hispanic	3 (7)
**Skin type, n (%)**		
	I. Very sun sensitive	25 (60)
	II. Average sun sensitive	17 (40)
	III. Low sun sensitive	0 (0)
**Family history of skin cancer, n (%)**		
	No	21 (50)
	Yes	21 (50)
**Personal history of skin cancer, n (%)**		
	No	42 (100)
	Yes	0 (0)

### Baseline Knowledge and History of Sunburn and Sun Protection in the Preceding 28 Days

Participants were asked to identify the characteristics of sunburns among the 6 offered (pink skin, red skin, pain, peeling, blistering, and skin hot to the touch). A total of 50% (22/44) of the participants were able to identify all 6 characteristics (range 1-6), with the most commonly overlooked characteristic being blistering. This was missed by 30% (13/44) of the participants.

Knowledge of sun strength and protection was tested by asking a series of questions regarding when the sun was strongest (time and month), UV rays, and clothing protection. On a scale of 0-6, scores ranged from 2 to 6, with 43% (18/42) of the participants scoring 2-3, 36% (15/42) scoring a 4, and 21% (9/42) scoring 5-6. All participants were able to recognize that the sun was strongest from 10 AM to 4 PM and that the best head protection was a hat with a 4-inch brim and neck flap. In contrast, 66% (29/44) and 84% (37/44) of the participants were able to identify the danger of UV-A and UV-B light, respectively; only 20% (9/44) of the participants recognized that the sun was strong enough to burn as early as March.

Among the 42 participants compared for getting a sunburn preceding and during the study, 71% (30) reported having sunburns in the 28 days preceding the study. Participants reported being outdoors for 31 minutes to 1 hour (26/42, 62%), more than 1 hour and up to 2 hours (11/42, 26%), and more than 2 hours and up to 3 hours (5/42, 12%) in the 28 days preceding the study. Frequency was reported as often or sometimes by 64% (27/42) of participants wearing sunscreen, 52% (22/42) wearing a shirt with sleeves, 21% (9/42) wearing a hat with a brim, and 71% (30/42) seeking shade. Wearing sunglasses was always done by 26% (11/42) and often or sometimes by 35% (15/42).

### Characterization of Sunburn and Sun Exposure During the 3 Periods

Sunburns were consistently experienced during all 3 periods. Although the mean UV-B exposure over the 3 periods declined, the differences were not statistically significant (Table 2). The mean proportion of each day spent outdoors by the sample demonstrated a statistically significant change in the 3 periods. During July and August, the ultraviolet index (UVI) was between 7 and 10, depending on the geographic location and local weather conditions [21,22]. This UVI was sufficient to produce sunburns in unprotected skin. Sunburn was most often experienced by participants during exercise (OR 5.65, 95% CI 1.60-6.10) and relaxation (OR 3.69, 95% CI 1.03-4.67) and less often with walking (OR 1.36, 95% CI 0.39-1.07) than by those who did not exercise, walk, or have outdoor relaxation.

The 13 sunburned body regions were compared 28 days before and during the 28 days of the study. Sunburns were experienced during the study by fewer subjects (n=18) than in the 28 days preceding the study (n=30). A statistically significant reduction in sunburn was on the shoulders and chest. (Table 4). The face was the most commonly sunburned area among the 13 body locations, with 52% (22/42) of sunburns before the study and 45% (19/42) during the study. Other body regions with sunburn reduction trends were the scalp and the back.

**Table 4:** Body region sunburned before and during the study for a sample of 42 participants.

Body regions	Participants sunburned before the study, n (%)	Participants sunburned during the study, n (%)	*P* value^a^
Face	22 (52)	19 (45)	.65
Neck	11 (26)	9 (21)	.68
Ears	4 (10)	3 (7)	.99
Scalp	5 (12)	1 (2)	.13
Shoulders	20 (48)	9 (21)	.01^b^
Back	9 (21)	4 (10)	.23
Chest	14 (33)	6 (14)	.02^b^
Stomach	4 (10)	0 (0)	—^c^
Arms	13 (31)	17 (41)	.45
Hands	3 (7)	5 (12)	.68
Buttocks	1 (2)	1 (2)	.99
Legs	8 (19)	8 (19)	.99
Feet	7 (17)	5 (12)	.77

^a^*P* values are from the McNemar test of paired proportions. As this was an exploratory analysis, no correction for multiple comparisons was made for the type I error rate.

^b^Shoulders and chest had significant decrease in sunburn.

^c^Not available. Owing to a limited sample size, the test could not be performed.

### Sun Exposure During Outdoor Activities

**Figure 4:**
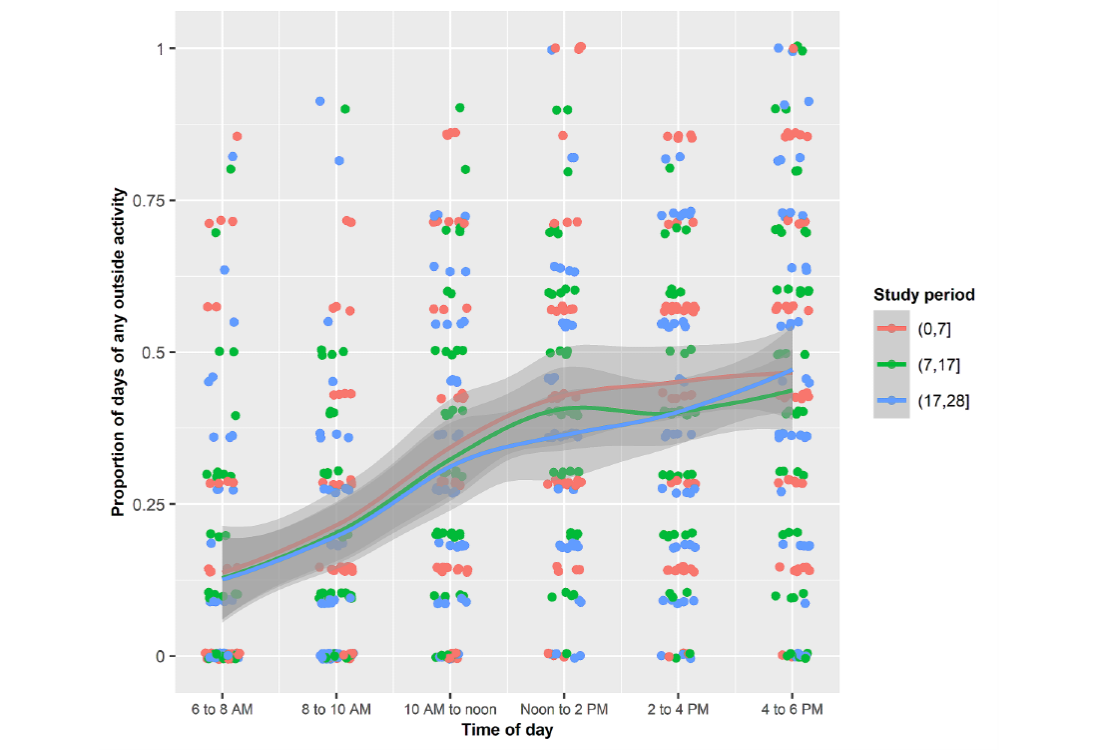
Mean proportions of each of the days spent outside by the sample for any activity during the 3 periods stratified by 2-hour periods.

**Figure 5:**
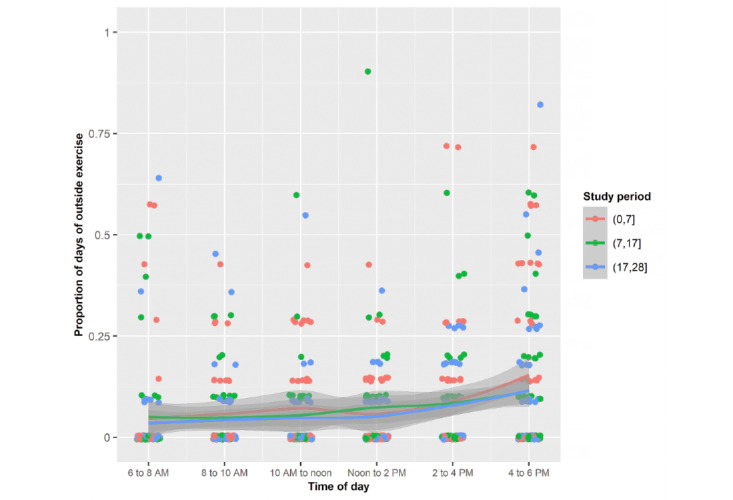
Mean proportions of each of the days spent outside by the sample for exercise during the 3 periods stratified by 2-hour periods.

**Figure 6:**
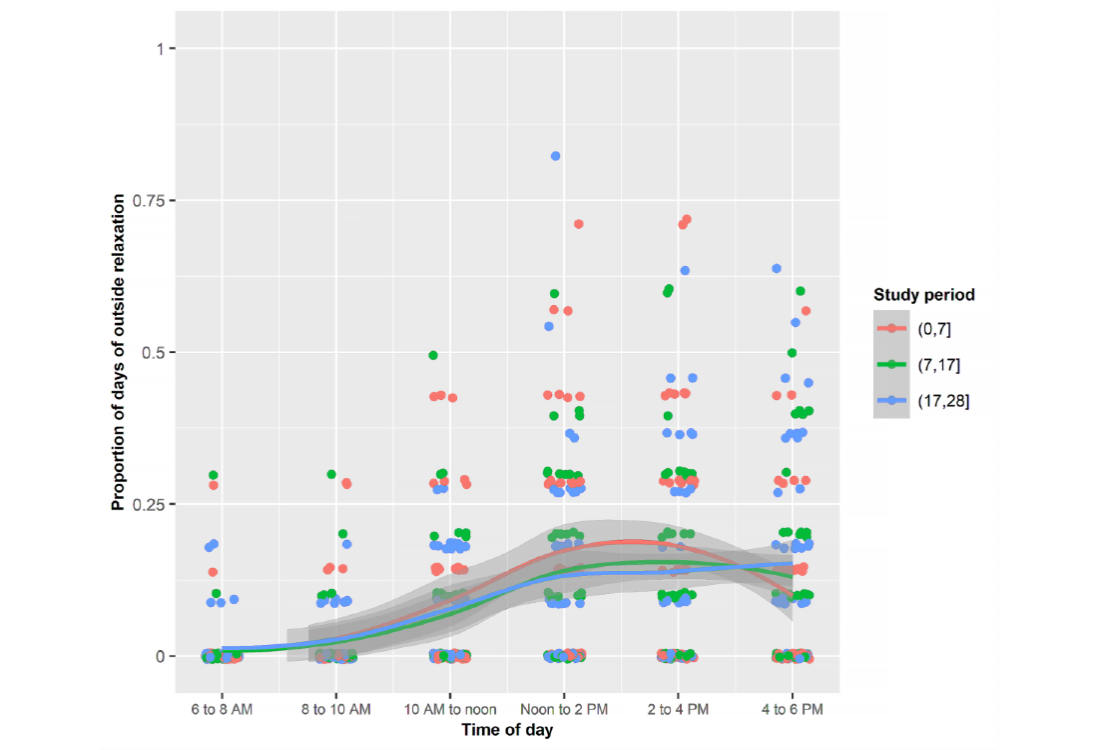
Mean proportions of each of the days spent outside by the sample relaxing during the 3 periods stratified by 2-hour periods.

**Figure 7:**
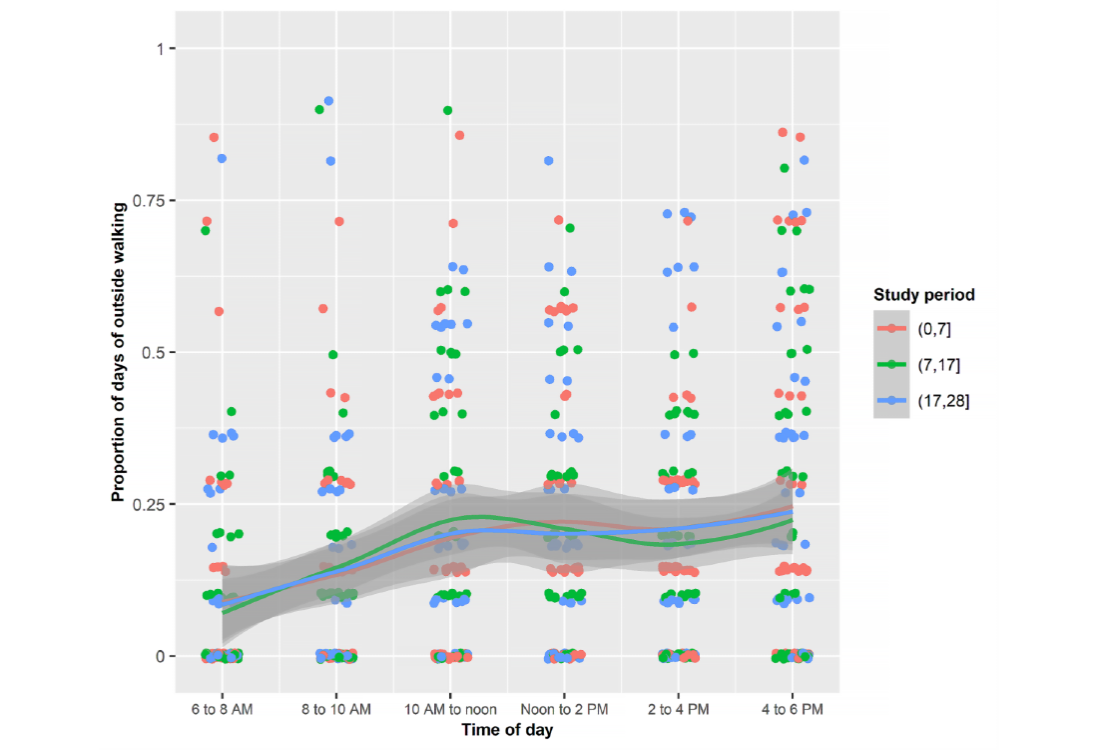
Mean proportions of each of the days spent outside by the sample walking during the 3 periods stratified by 2-hour periods.

Young adults were most often outdoors exercising from 2 to 6 PM, walking from 10 AM to 6 PM, and relaxing from noon to 2 PM (Figures 4-7). Public health strategies during the COVID-19 pandemic in the United States restricted team sports and closed gyms. Shelter-in-place policies asked workers to perform their jobs from home. There was no significant difference in the proportion of time devoted to these outdoor activities during the 3 periods.

**Figure 8:**
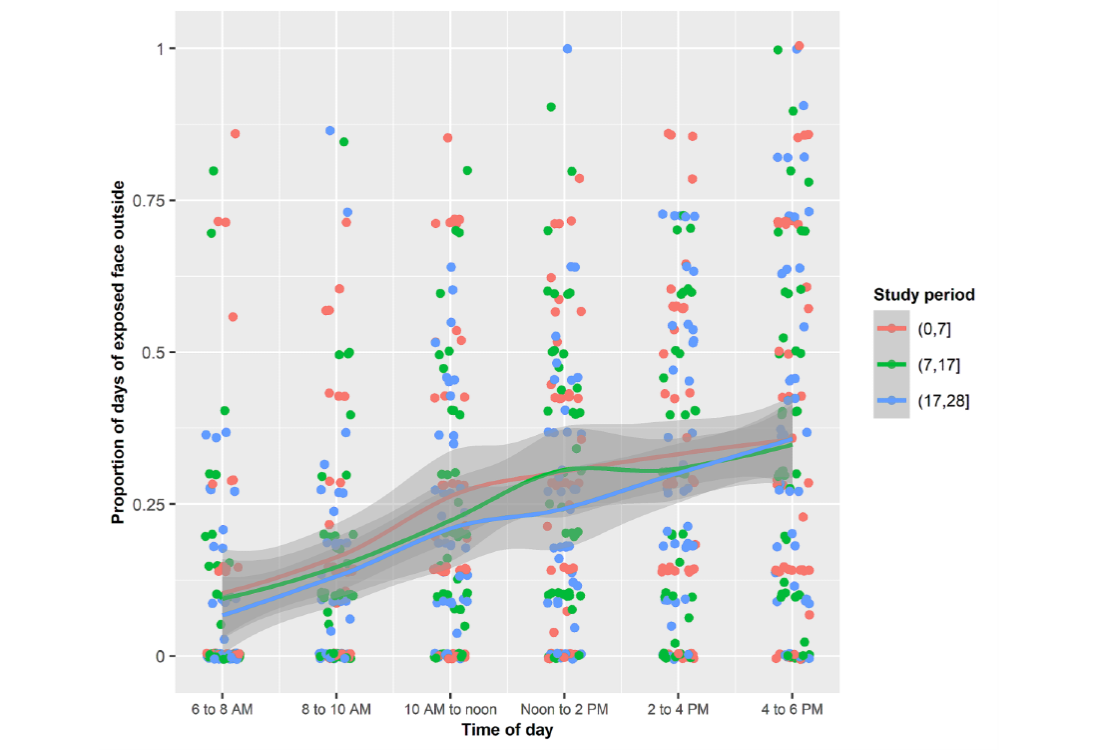
Mean proportions of each of the days spent outside by the sample with an unprotected face during the 3 periods stratified by 2-hour periods.

In all 3 periods, the proportion of the day spent outside with an unprotected face was greatest from noon to 6 PM, which resulted in facial sunburn (Figure 8).

### Sun Protection

Sunscreen was the principal form of sun protection used, with 73% (31/42) of participants reporting applying it each day when they went outside before noon (24/42, 58%) and in the afternoon (18/42, 42%). Most participants used one type of sunscreen (37/42, 88%) with an SPF 15-49 (23/42, 55%) or SPF 50+ (18/42, 43%). Sunscreen was mostly applied to the face (38/42, 91%) and was reapplied to the face in the afternoon by 10% (4/42) of participants. The statistically significant reduction in shoulder and chest sunburns may be attributed to wearing a T-shirt that covered the shoulders (Table 5).

**Table 5:** Body regions protected during the study with sunscreen and clothing.

Body regions	Sunscreen applied (n=265), n (%)	Clothing worn (n=124), n (%)
Face	242 (91)	14 (11)
Neck	99 (37)	2 (2)
Ears	63 (24)	7 (6)
Scalp	6 (2)	24 (19)
Shoulders	41 (16)	102 (82)
Back	22 (8)	106 (86)
Chest	14 (44)	88 (71)
Stomach	31 (12)	106 (86)
Arms	80 (30)	21 (17)
Hands	51 (19)	7 (6)
Buttocks	5 (2)	85 (69)
Legs	48 (18)	42 (34)
Feet	21 (8)	62 (50)

### Goals Selected

The most commonly selected goal was to wear a shirt that “covers my shoulders when I am outdoors” (25/42, 60%). Other goals were selected less frequently, as follows: (1) “Plan my outdoor activities to avoid being outside from 10 AM to 2 PM” (13/42, 31%), (2) “Apply sunscreen before I go outdoors” (12/42, 29%), (3) “Wear a hat when I am outdoors” (9/42, 21%), (4) “Apply sunscreen to all of the areas of my body that may be exposed to the sun” (8/42, 19%), (5) “Be careful not to exceed the amount of UV my skin can tolerate” (8/42, 19%), and (6) “Pay attention to the strength of the sun by checking the UV Guard report 15 minutes after I go outside” (6/42, 14%).

The intention to continue to perform the sun protection behavior varied from 86% (36/42) reporting intending to “pay attention to the strength of the sun by checking the UV Guard report 15 min after I go outside,” 81% (34/42) intending to apply sunscreen to all of the areas of the body that may be exposed to the sun, 81% (34/42) intending to be careful not to exceed the amount of UV the skin can tolerate, 79% (33/42) intending to wear a hat when outdoors, 71% (30/42) intending to apply sunscreen before going outdoors, 69% (29/42) intending to plan outdoor activities to avoid being outside from 10 AM to 2 PM, and 41% (17/42) intending to wear a shirt that covers the shoulders when outdoors.

### Anxiety and Confidence

Anxiety about sun protection and sun exposure tended to decrease in the 3 periods from the mean score of 32.29 (SD 6.38) in period 1 to 31.69 (SD 8.84) in period 2 and to 31.95 (SD 8.41) in period 3; however, it was not statistically significant. Confidence in their ability to protect their skin from the sun and use sunscreen with reapplication, wear protective clothing, and seek shade when outdoors tended to increase in the 3 periods from the mean score of 37.33 (SD 4.72) in period 1 to 38.45 (SD 5.17) in period 2 and to 39.45 (SD 8.18) in period 3; however, this was not statistically significant.

### Exit Survey Themes

Participants were surveyed at the end of the study to evaluate their experiences with the UV Guard program. Most participants (26/42, 62%) felt that they spent less time outdoors this summer in comparison with last summer. This change in time outdoors was most commonly attributed to the COVID-19 pandemic and work.

Participants expressed interest in continuing their use of the UV sensor and the UV Guard app. A total of 69% (29/42) of the participants noted that they were either extremely or moderately willing to continue using the sun protection system as part of the research, and another 26% (11/42) reported that they were either somewhat or slightly willing to do so as well. In addition, 79% (33/42) of the participants were willing to continue using the sun protection system outside of a research setting. Among this sample of 33 users willing to continue personal use, 39% (13) would use it every day.

Participants were also asked about their sun exposure and protection habits over the course of the study. A total of 48% (20/42) of the patients noted a shift in the time they went outside to periods with less-intense UV exposure. Knowledge of real-time UV exposure, which was presented in the UV Guard app, also encouraged changes in users’ habits. In particular, 33% (14/42) of the participants changed the duration of the time spent outside. In addition, 38% (16/42) of the participants changed their use of clothing for sun protection or seeking shade based on their observed UV exposure. For example, some participants chose to wear hats and T-shirts when outside midday or sought shaded areas during outdoor jogging.

## Discussion

### Principal Findings

This proof-of-concept research demonstrated the feasibility of young adult participants using a sun protection system consisting of a personal UV-B dosimeter (UV Guard) providing real-time feedback about their personal UV exposure as graphs on their smartphone and receiving daily text messages for 28 summer days. The unique daily assessment of sunburns sustained in various regions of the body and sun protection usage on body regions made it possible to observe that sunburns of the shoulders and chest significantly decreased from the period before and after the study period, and the trend for sunburns of the arms and hands increased. Although the daily UV-B dose declined over the 3 periods, the reduction was not statistically significant. In exit surveys, 33% (14/42) of the participants reduced their cumulative daily UV-B exposure and 38% (16/42) increased their use of sun protection.

The mean daily UV-B dose during the 3 periods, which ranged from 91.96 J/m^2^ to 62.73 J/m^2^, exceeded the recommended daily occupational exposure limit of 30 J/m^2^ within an 8-hour time frame for sensitive, unprotected skin [23]. Thus, sunburn may be expected in body areas not protected by clothing or the application of sunscreen. Defining sunburn in the recruitment materials was an important initial step in improving awareness and reporting of sunburn [10]. The reduction in facial sunburns during the study compared with those during the period preceding the intervention was attributed to improved sunscreen usage, reduced scalp sunburn to wearing a baseball cap, and reduced shoulder sunburn to wearing a T-shirt that covered the shoulders. The median age of the participants in this study (22 years) may have contributed to the lack of improvement in sunburn on the arms and hands during the study. People in the age range of 18-24 years have difficulty diminishing risk-taking behaviors, such as unprotected exposure to UV [24]. Interventions in this age group such as attempts to stop binge drinking and unsafe sex have an underwhelming track record.

Although daily tracking of sun protection behavior was repetitive, the repetition may have facilitated cognitive processing and the development of healthy habits. The daily surveys provided line drawings of types of clothing and explained the amount of sun protection provided, thus increasing knowledge and awareness of sun protection. The daily text messages initially provided knowledge about the types of sun protection and times of the day when sun protection was most necessary. Daily messages were received at the most advantageous time selected by young adults (11 AM). Working from home during the pandemic may have skewed the selection of the time of the day to receive text messages. Young adults may have selected 11 AM as the time to receive the text as the proportion of participants going outdoors increased from 25% to 50% from 10 AM to noon. Without the constraints of the pandemic, others may prefer receiving messages earlier in the day before leaving home.

### Limitations

Although this study has several strengths, response bias may have occurred, resulting in the overestimation of sun protection use. The effects of social desirability bias are expected to be minimal because of remote recruitment, provision of the intervention, and data collection. Initiation and maintenance of health promotion behaviors such as outdoor exercise and sun protection were disrupted by COVID-19, which required social-distancing policies and stay-at-home practices. Thus, the sun exposure and protection reported by the participants in this study may not be generalizable after cessation of the COVID-19 policies.

Technical limitations in the design of the UV Guard app and dosimeter may have contributed to less definitive results. As the device relies on Bluetooth communication with the UV Guard app, connectivity was hindered when the dosimeter was outside the range of the user’s phone. Some participants reported that they did not carry their phones with them during outdoor exercise. In these instances, users may not have been informed with a real-time measure of sun exposure.

In addition, the sun protection system aimed to warn users as they approached their sunburn thresholds. However, participants did not receive push notifications from the UV Guard app to alert them of this risk. Rather, users were required to manually open the app to view their real-time UV exposure.

### Comparison With Prior Work

Earlier efforts to reduce unprotected UV exposure among young adults focused on (1) a single point-in-time intervention such as counseling during group meetings and providing print material with appearance-focused messages to promote sun protection [25]; (2) physician counseling, including appearance-focused messages stressing the aging effects of UV on the skin and skin cancer prevention [26]; or (3) the personalization of the risk of UV exposure with a UV photograph of the participants’ face showing photodamage, which resulted in increased sun protection among young adults 4-5 months and 12 months later [27]. As smartphones became ubiquitous, apps provided individuals with tailored data about their UV exposure risk based on skin sun sensitivity, including current and forecasted UVIs (Solar Cell [Klein Buendel, Inc]) [28], Healthy Texts (Cancer Australia) [29,30], and UV4.me (Rutgers Cancer Institute of New Jersey) [31]. In the 7-week interim analysis of SolarCell, there was an increase in the use of wide-brimmed hats among younger app users (24% vs 17%; *P*=.045), but the trend did not remain significant by the 12-week posttest analysis [32]. The approach of using a UVI based on weather predictions in a geographic region may have been impaired by people’s poor overall comprehension of the UVI [33]. Studies using similar technology-based tailored text messages improved sun protection behaviors in the population studied [29,30,34].

Personal UV dosimeters and real-time UV detection communicated to users with smartphones allow interventions to be delivered by eHealth. An advantage of delivering health promotion via these mobile devices was that an already existing infrastructure was used. The Hacker et al [8] study randomized participants received either (1) the SunSmart app (Cancer Council, Victoria), (2) the UV dosimeter (Healthtronics SunSafe Pty Ltd) providing feedback set to their skin type, or (3) control with no intervention. Participants completed daily sun diaries for 4 weeks. Users received a warning when their UV-B exposure approached their sunburn threshold. The randomized controlled trial by Hacker et al [8], which gave the user their personal real-time UV dose, demonstrated a reduction in unprotected time among those using UV monitors. Our research provided an app, daily text messages, and UV dosimeter with real-time personal UV dose; therefore, relevance to the user may have been enhanced by the text messages, daily surveys, and real-time personal UV exposure provided with this high-intensity daily intervention. To our knowledge, no other trial has performed daily assessment of sunburn sustained in various regions of the body and aligned this with self-reported sun protection at 2-hour intervals. Although participants in the Hacker et al [35] study completed daily sun diaries, sun protection was recorded in less precise intervals and sunburn locations were not specified. In addition, the nature of participants’ outdoor activities was not recorded in detail.

Although self-monitoring devices to assess disease treatment adherence are widespread, primary prevention behavioral change interventions are less prevalent and usually focus on changing nutrition and physical activity [36]. Evaluation of the effectiveness of physical activity and nutrition interventions has been limited by short follow-up periods and lack of patient-important primary outcomes. Although intention to change behavior and behavioral changes may be initiated during high-intensity intervention, long-term maintenance of sun protection behaviors may not be sustainable. In some regions of the United States, seasonal changes in UV intensity limit the need for sun protection in winter, thus interrupting daily reinforcement of sun protection habits. Furthermore, preventive behaviors are impacted differently because of their underlying motivations, for example, sunscreen use is influenced by social norms and attitudes [37]. Sun exposure may be related to an individual’s daily routine activities, such as sitting outside to eat lunch. Our hypothesis is that as participants perform customary outdoor activities, they have sun protection habits associated with those routine activities; therefore, they will be less likely to get sunburn during routine outdoor activities. When participants sporadically engage in outdoor activities, sun protection will not be used because the habit is not established for the sporadic activity and sunburn occurs. It may not be realistic to expect sun protection and the reduction of sun exposure sustained during sporadic outdoor activities by young adults to be facilitated by mobile health with wearable sensors and mobile technologies.

### Conclusions

This study demonstrated the feasibility of providing real-time UV-B exposure relative to the participants’ anticipated sunburn threshold. Exit interviews and a statistically significant reduction in shoulder and chest sunburn, as well as trends in reducing sunburn of the scalp, and back indicated that the UV Guard program improved shifting outdoor activities to periods with less-intense UV exposure and wearing baseball caps and T-shirts among some young adults. Although feasibility was demonstrated, the UV Guard program needed to be optimized by having the app provide in-the-moment alarms of impending sunburn communicated by a buzzer or voice message to the user’s smartphone or incorporated into the device as a vibratory, audible, or light signal. The time required to cross the sunburn threshold needs to be adjusted for the daily report of sunscreen application and clothing worn.

This study was intended to prevent sunburn and improve sun protection by providing knowledge-based daily tips and suggestions for goal setting that promote self-efficacy. Future research will explore whether young adults use the feedback provided by real-time personal UV dosimeters to stay below their daily sunburn threshold with the unintended consequence of increasing their weekly cumulative UV exposure, which is associated with increased photoaging and skin cancer. Furthermore, in the era of the COVID-19 pandemic, patterns of health behavior have been disrupted by social-distancing and sheltering-in-place policies. Future sun protection research among young adults with sun-sensitive skin will need to be postponed until the COVID-19 policies abate.

## Appendix

Multimedia Appendix 1 The web-based consent provided to participants.

Multimedia Appendix 2 The schedule of measures provided to participants during the 28-day study and the web-based measures used in the study. The web-based measures are (1) baseline measures, including demographic responses; the recall of sun exposure, sunburn experienced, and sun protection used in the prior 28 days; and knowledge of sun intensity and protection; (2) measures repeated at intervals, including anxiety and confidence and daily sun habits, midpoint goal setting, and the follow-up of intentions to perform goals; and (3) exit items, including the system usability scale, questions comparing the summer experience of going outdoors in 2019 and 2020, the use of UV sensors during the study, and the willingness to continue to use the UV sensor and UV Guard app.

## Abbreviations

MED: minimal erythema dose
OR: odds ratio
REDCap: Research Electronic Data Capture
SPF: sun protection factor
UVI: ultraviolet index
