# Supplementary material for: Real-Time UV Measurement With a Sun Protection System for Warning Young Adults About Sunburn: Prospective Cohort Study
Source: JMIR Mhealth Uhealth. 2021 May 6;9(5):e25895. doi: 10.2196/25895 (PMC8138709; doi:10.2196/25895)
Supplement: Multimedia Appendix 1 [file mhealth_v9i5e25895_app1.pdf]

Consent

Please complete the survey below.

Thank you!

Title of Research Study: A real-time, cost-effective, accurate UV measurement and sun protection system to prevent and reduce the incidence of sunburn in high-risk consumers.(SBIR R44, to Wearifi, Inc. R44CA224658-01)

Investigator: June K. Robinson, MD

Supported By: This research is supported by the National Institutes of Health.

Key Information:

The first few pages of this document include a summary of this study to help you decide whether or not to participate. Detailed information is provided after the summary.

Why am I being asked to take part in this research study?

We are asking you to take part in this research study because you are between 18-39 years old and have skin that can get pink a day after being in the sun, sunburn.

What should I know about a research study?

Someone will explain this research study to you.

Whether or not you take part is up to you.

You can choose not to take part.

You can agree to take part and later change your mind.

Your decision will not be held against you.

You can ask all the questions you want before you decide.

Why is this research being done?

The purpose of this study is to prevent ultraviolet light (UV) overexposure and sunburns in at-risk young adults by providing people real-time UV exposure information. This real-time UV information is obtained by a wearable UV sensor that registers sunlight exposure. The amount of sunlight exposure is seen using the corresponding smartphone application. The miniature sensor is about the size of a nickel and is worn like a wristwatch. The main benefit is becoming more aware of your sun exposure and sun protection habits.

How long will the research last and what will I need to do?

We expect that you will be in this research study for 30 days. You will be asked to wear the UV sensor and transmit data for 28 days and complete daily surveys online. Text message reminders to complete surveys and transmit data will be sent to your personal phone multiple times each day.

More detailed information about the study procedures can be found under the section What happens if I say “Yes, I want to be in this research”?

Will being in this study help me any way?

We cannot promise any benefits to you or others from your taking part in this research. However, possible benefits include becoming more aware of your sun exposure and protection habits.

What happens if I do not want to be in this research?

Participation in research is completely voluntary. You decide whether or not to participate. If you choose to not participate, there will be no penalty to you or loss of benefit to which you are entitled.

Your alternative to participating in this research study is to not participate.

Detailed Information: The rest of this document includes detailed information about this study (in addition to the information listed above).

Whom can I talk to?

If you have questions, concerns, or complaints, or think the research has hurt you, talk to the research team. You may send an email with your request and provide a telephone number for the principal investigator to call you. The contact information for the principal investigator is:

June K. Robinson, MD (office hours: 9 AM – 5 PM)

june-robinson@northwestern.edu

This research has been reviewed and approved by an Institutional Review Board (IRB). You may talk to them at (312) 503-9338 or irb@northwestern.edu if:

Your questions, concerns, or complaints are not being answered by the research team

You cannot reach the research team

You want to talk to someone besides the research team

You have questions about your rights as a research participant

You want to get information or provide input about this research.

How many people will be studied?

We expect about 40 people will be in this research study.

What happens if I say “Yes, I want to be in this research”?

If you agree to participate in this research study, you will receive an email with a baseline survey is housed in Northwestern’s secure database, REDCap. The survey will capture your contact information, demographics, and assess your knowledge about the sun’s strength. The wrist worn sun sensor will be mailed to your home address. After you receive the sun sensor, you may have an initial telephone call with a member of the research team to assist you with downloading the app to your phone. The survey will capture your contact information, demographics, and assess your knowledge about the sun’s strength. For 28 days of the study, you will be expected to wear the sun sensor while awake and data will be transmitted to your phone from the sensor.

For the 28 days of wearing the sensor, you will also complete an online survey about your sun protection. In addition, we ask you to continue your usual outdoor activity behavior by spending around an hour outdoors between 8AM and 5 PM and of that hour, about 30 minutes will be consecutively spent outdoors. The daily survey will arrive in your email at approximately 6 PM.

At day 17, you will receive the same daily survey, but will answer additional questions regarding goals to improve your sun protection behavior.

Within 2-3 days of completing your 28 days of data transmission, you will return the sensor in a pre-paid envelop.

What are my responsibilities if I take part in this research?

If you take part in this research, you will be responsible for completing the daily research surveys and wearing your sun sensor.

What happens if I say “Yes”, but I change my mind later?

You can leave the research at any time; it will not be held against you.

If you decide to leave the research, contact the investigator so that the investigator can obtain your permission to use any data collected, up until that point, to be used in research.

Choosing not to be in this study or to stop being in this study will not result in any penalty to you or loss of benefit to which you are entitled. Specifically, your choice not to be in this study will not negatively affect your right to any present or future medical treatment, your class standing (for students enrolled in a class at NU), or your present or future employment (for employees at NU or its affiliates).

Detailed Risks: Is there any way being in this study could be bad for me?

This study involves the use of your identifiable, personal information and there is a chance that a loss of confidentiality could occur. The researchers have procedures in place to lessen the possibility of this happening. See the section below titled: “What happens to the information collected for the research?”.

Will it cost me anything to participate in this research study?

Taking part in this research study will not lead to any costs to you.

Will being in this study help me in any way?

We cannot promise any benefits to you or others from your taking part in this research. However, possible benefits include becoming more aware of your sun exposure and protection habits.

What happens to the information collected for the research?

Efforts will be made to limit the use and disclosure of your personal information, including research study and medical records, to people who have a need to review this information. We cannot promise complete secrecy. Organizations that may inspect and copy your information include the IRB and other representatives of this institution.

To help us protect your privacy, we have obtained a Certificate of Confidentiality from the National Institutes of Health (NIH). The researchers can use this Certificate to legally refuse to disclose information that may identify you in any federal, state, or local civil, criminal, administrative, legislative, or other proceedings; for example, if there is a court subpoena. The researchers will use the Certificate to resist any demands for information that would identify you. You should understand that a Certificate of Confidentiality does not prevent you or a member of your family from voluntarily releasing information about yourself or your involvement in this research. If an insurer, medical care provider, or other person obtains your written consent to receive research information, then the researchers will not use the Certificate to withhold that information.

The sponsor, monitors, auditors, the IRB, the Northwestern University Office for Research Integrity, the US Office of Research Integrity (ORI), the US Office for the Protection of Human Research Protections (OHRP), and the US Food and Drug Administration (FDA) may be granted direct access to your medical records to conduct and oversee the research. By signing this document, you are authorizing this access. We may publish the results of this research. However, we will keep your name and other identifying information confidential.

A description of this clinical trial, NCT03344796, will be available on http://www.ClinicalTrials.gov, as required by U.S. Law. This Web site will not include information that can identify you. At most, the Web site will include a summary of the results. You can search this Web site at any time.

Data Sharing

De-identified data from this study may be shared with the research community at large to advance science and health. We will remove or code any personal information that could identify you before files are shared with other researchers to ensure that, by current scientific standards and known methods, no one will be able to identify you from the information we share. Despite these measures, we cannot guarantee anonymity of your personal data.

What else do I need to know?

If you agree to take part in this research study, we will pay you $200 for your time and effort in the form of an Amazon electronic gift card.

This consent expires on 12/31/2021. After this date, Northwestern University may not gather new information about you, use or disclose your personal health information collected in this study for any purpose other than the research study described in this consent unless Northwestern University obtains permission to do so from you. Illinois State Law permits use and disclosure of your mental health information only to the extent specified in this document.

During this study, you may be coming to a Northwestern Memorial Healthcare Corporation entity (for example, Northwestern Memorial Hospital, Prentice Women's Hospital) for research appointments or to get clinical services, such as lab tests, needed for the study. When that happens, you will be scheduled for these services through the NMHC computer system. When a clinical exam or lab is done by NMHC or one of its employees for the purpose of this research study, that information will be kept in both NMHC's clinical records and in the study records.

The following clinical providers may give the researchers information about you: all current and previous health care providers, including but not limited to the Shirley Ryan AbilityLab (SRALAB), Northwestern Medical Group (NMG), Northwestern Memorial Hospital (NMH), and Northwestern Lake Forest Hospital (NLFH).

Once we have the health information listed above, we may share some of this information with the following offices or entities outside of Northwestern University and its clinical partners (or affiliates): the Northwestern University Institutional Review Board Office and Office for Research Integrity; the US Office of Research Integrity; the US Office for Human Research Protections; the US Food and Drug Administration.

Any research information shared with outside entities will not contain your name, address, telephone or social security number or any other personal identifier unless disclosure of the identifier is necessary for review by such parties or is required by law or University policy [except that such information may be viewed by the Study sponsor and its partners or contractors at the Principal Investigator's office].

The following entities may receive your health information:

Authorized members of the Northwestern University workforce, who may need to see your information, such as administrative staff members from the Office for Research, Office for Research Integrity and members of the Institutional Review Board

Clinical affiliates, including but not limited to the Shirley Ryan AbilityLab (SRALAB), Northwestern Medical Group (NMG), Northwestern Memorial Hospital (NMH), Northwestern Lake Forest Hospital (NLFH), and the Ann & Robert H. Lurie Children's Hospital of Chicago (Lurie Children's). Your participation in this clinical trial may be tracked in an

electronic database and may be seen by investigators running other trials that you are enrolled in and by your healthcare providers.

Clinical affiliates, including but not limited to Northwestern Medical Group (NMG), Northwestern Memorial Hospital (NMH), and Northwestern Lake Forest Hospital (NLFH), for purposes including, but not limited to, the affiliate's provision of care to you and/or the affiliate's scheduling of appointments and/or billing activities

Other University research centers and University contractors who are also working on the study

Study monitors and auditors who make sure that the study is being done properly

Government agencies and public health authorities, such as the Food and Drug Administration (FDA) and the Department of Health and Human Services (DHHS).

Those persons who get your health information may not be required by Federal privacy laws (such as the Privacy Rule) to protect it. Some of those persons may be able to share your information with others without your separate permission.

The results of this study may also be used for teaching, publications, or for presentation at scientific meetings.

Although you may revoke consent to participation in this research at any time and in any format, you must revoke authorization for use or disclosure of your health information in writing. To revoke your authorization, write to:

PI's Name: June K. Robinson, MD

Institution: Northwestern University

Department: Dermatology

Address: 645 N Michigan Ave., Ste 1050

Chicago, IL 60611

You do not have to authorize the use or disclosure of your health information; however, you will not be allowed to take part in this research study. If you do not authorize the use or disclosure of your health information, it will not affect your treatment by health care providers, or the payment or enrollment in any health plans, or affect your eligibility for benefits.

A copy of this signed consent document, information about this study, and the results of any test or procedure done may be included in your medical records and may be seen by your insurance company.

Optional Elements:

The following research activities are optional, meaning that you do not have to agree to them in order to participate in the research study. Please indicate your willingness to participate in these optional activities by placing your initials next to each activity.

1) The researcher may audio record me to aid with data analysis. The researcher will not share these recordings with anyone outside of the immediate study team.

◯ I agree
◯ I disagree

2) The researcher may contact me in the future to see whether I am interested in participating in other research studies by the Principal Investigator of this study.

◯ I agree
◯ I disagree

Consent

3) If you want a copy of this consent for your records, you can print it from the screen.

If you wish to participate, please click the “I Agree” button and you will be taken to the survey.

If you select “I Agree,” you will be required to type your first and last name, the date, and provide your electronic signature using the cursor.

If you do not wish to participate in this study, please select “I Disagree” or select X in the corner of your browser.

◯ I Agree
◯ I Disagree

4) First Name ________________________

5) Last Name ________________________

6) Today's date ________________________

7) Please sign your full name ______________________________
