# Supplementary material for: Real-Time UV Measurement With a Sun Protection System for Warning Young Adults About Sunburn: Prospective Cohort Study
Source: JMIR Mhealth Uhealth. 2021 May 6;9(5):e25895. doi: 10.2196/25895 (PMC8138709; doi:10.2196/25895)
Supplement: Multimedia Appendix 2 [file mhealth_v9i5e25895_app2.pdf]

Schedule of Measures for Young Adult Participants

Measure	**Day 0** Baseline	**Days 0-7** Observe	**Days 8-17** Daily text messages	**Day 18** Structured goal setting	**Days 18-28** Daily text messages
**Self-Report**					
Demographics	x				
Sunburn in last 28 days	x				
Confidence and Anxiety	x	D7	D16		D28
Daily sunburn/ sun protection		x	x	x	x
Structured goal				x	
System Usability Scale					x
Willingness to continue use					x
**Sensor**					
Daily UV		x	x	x	x
**Intervention**					
Daily text messages			x	x	x
UV exposure visualization		x	x	x	x

Baseline Questionnaire

Please complete the survey below.

Thank you!

Phone number	__________________
Email	__________________
Address	__________________
City	__________________
State (Abbreviation)	__________________
Zip Code (postal code)	__________________
Time Zone	◯ Eastern Daylight Time (NY, GMT-4)<br>◯ Central Daylight Time (IL, GMT-5)<br>◯ Mountain Daylight Time (CO, GMT-6)<br>◯ Mountain Standard Time (AZ, GMT-7)<br>◯ Pacific Daylight Time (CA, GMT-7)<br>◯ Alaska Daylight Time (AL, GMT-8)<br>◯ Hawaii-Aleutian Standard Time (HI, GMT-10)
Please provide the first and last name of your emergency contact	__________________
Please provide the phone number of the emergency contact that you listed above	__________________

Demographic Information

What is your gender?	◯ Male<br>◯ Female<br>◯ Prefer not to answer
What is your birth date?	__________________
Your age:	__________________
Are you of Spanish and/or Latino origin?	◯ Yes ◯ No

How do you describe your race?

- ◯ American Indian or Alaska Native
- ◯ Asian
- ◯ Black or African American
- ◯ Native Hawaiian or Other Pacific Islander
- ◯ White
- ◯ Unknown
- ◯ Other
- ◯ Prefer not to answer

Sun Sensitivity and Skin Cancer History Questions

Please complete the following sentence by selecting all answers that apply.

A sunburn can be
(Please select all that apply)

- ☐ Pink skin
- ☐ Very red skin
- ☐ Pain
- ☐ Peeling
- ☐ Blistering
- ☐ Hot to touch

Has someone in your family had skin cancer in the past?

◯ Yes ◯ No

Have you had skin cancer in the past?

◯ Yes ◯ No

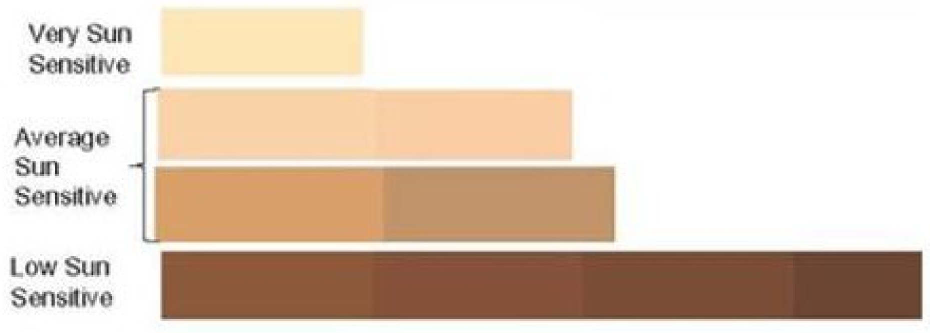

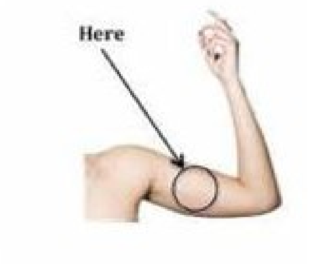

Based on the image shown above, what is the color of the untanned skin on your upper inner arm?

- ◯ a. Very Sun Sensitive
- ◯ b. Average Sun Sensitive
- ◯ c. Low Sun Sensitive

Did you get a SUNBURN in the past 28 days on even a small part of your skin?

◯ Yes ◯ No

What part or parts of your body got a SUNBURN in the past 28 days: (select all that apply)	☐ Scalp<br>☐ Face<br>☐ Ears<br>☐ Neck<br>☐ Shoulders<br>☐ Back<br>☐ Chest<br>☐ Stomach<br>☐ Arms<br>☐ Hands<br>☐ Buttocks<br>☐ Legs<br>☐ Feet
In the past 28 days, on average, how many hours are you outside per day between 10 AM and 4 PM on WEEKDAYS (Monday-Friday)? Select one answer.	◯ 30 minutes or less<br>◯ 31 minutes to 1 hour<br>◯ More than 1 hour and up to 2 hours<br>◯ More than 2 hours and up to 3 hours<br>◯ More than 3 hours and up to 4 hours<br>◯ More than 4 hours and up to 5 hours<br>◯ More than 5 hours
In the past 28 days, on average, how many hours are you outside per day between 10 AM and 4 PM on WEEKEND DAYS (Saturday & Sunday)? Select one answer.	◯ 30 minutes or less<br>◯ 31 minutes to 1 hour<br>◯ More than 1 hour and up to 2 hours<br>◯ More than 2 hours and up to 3 hours<br>◯ More than 3 hours and up to 4 hours<br>◯ More than 4 hours and up to 5 hours<br>◯ More than 5 hours
In the past 28 days, how many times did you get a sunburn (a small part of your skin turned pink) for more than 12 hours? Select one answer.	◯ 0<br>◯ 1<br>◯ 2<br>◯ 3<br>◯ 4<br>◯ 5 or more
In the past 28 days, how many times did you get a sunburn (a small part of your skin was sensitive to touch) for more than 12 hours? Select one answer.	◯ 0<br>◯ 1<br>◯ 2<br>◯ 3<br>◯ 4<br>◯ 5 or more

For the following questions, think about what you would normally do when you are outside on a warm sunny summer day. Select one answer for each item.

	Always	Often	Sometimes	Rarely	Never
How often do you wear ○ SUNSCREEN?	○	○	○	○	
How often do you wear a SHIRT ○ WITH SLEEVES that cover your shoulders?	○	○	○	○	
How often do you wear a HAT ○ with a brim?	○	○	○	○	
How often do you stay in the ○ SHADE or UNDER AN UMBRELLA?	○	○	○	○	
How often do you wear SUNGLASSES?	○	○	○	○	○

For the following questions, think about what you would normally do when you are outside on a CLOUDY summer day. Select one answer for each item.

	Always	Often	Sometimes	Rarely	Never
How often do you wear ○ SUNSCREEN?	○	○	○	○	
How often do you wear a SHIRT ○ WITH SLEEVES that cover your shoulders?	○	○	○	○	
How often do you wear a HAT ○ with a brim?	○	○	○	○	
How often do you stay in the ○ SHADE or UNDER AN UMBRELLA?	○	○	○	○	
How often do you wear SUNGLASSES?	○	○	○	○	○

What is the number of the sun protection factor (SPF) of the sunscreen that you usually use?

- ○ I do know: SPF 1-10
- ○ I do know: SPF 11-15
- ○ I do know: SPF 16-30
- ○ I do know: SPF greater than 30
- ○ I do not know
- ○ I do not wear sunscreen

Please estimate how often you purposely spend time in the sun in order to get a tan? Select one answer.

- ○ Never
- ○ Rarely
- ○ Sometimes
- ○ Often
- ○ Always

How many times in the last 3 months have you used a tanning bed or booth with tanning lamps? Select one answer. Select one answer.

- ○ Never
- ○ Once
- ○ Two or three times
- ○ Four or five times
- ○ Six to ten times
- ○ More than 10 times

Do you like sitting out in the sun?	◯ Yes ◯ No
Why do you like sitting in the sun?<br>(Please check all that apply)	☐ Warmth<br>☐ Vitamin D<br>☐ Relaxation<br>☐ To get a tan

Knowledge of sun strength and sun protection

The strength of the sun depends on which of the following factors?<br><br>(Select all that apply)	☐ Time of day<br>☐ Season of the year<br>☐ Latitude<br>☐ Cloud cover
In the greater Chicago area, when does the strength of the sun startsto increase?	◯ March<br>◯ April<br>◯ May<br>◯ June<br>◯ July
The sun's energy is called ultraviolet (UV) radiation or ultraviolet (UV) light. There are three types of UV rays.<br><br>Which types of Ultraviolet (UV) light can cause skin cancer?<br>(Select all that apply)	☐ Ultraviolet A<br>☐ Ultraviolet B<br>☐ Ultraviolet C
The strength of the sun is strongest during what hours of the day?	◯ Between 10 AM and 4 PM<br>◯ Between 6 AM and 4 PM<br>◯ Between 10 AM and 12 PM
What kind of hat provides the best protection from the sun?	◯ Baseball cap<br>◯ 4-inch brim hat<br>◯ 4-inch brim hat with a sun protective neck flat cover
Do sunglasses need to say 100% UV protection or UV400 to protect the eyes from UVA and UVB?	◯ Yes<br>◯ No
To protect the face against the sun, an adult needs an average of how much sunscreen?	◯ 1 tablespoon<br>◯ 1 teaspoon<br>◯ 1/3 teaspoon<br>◯ 1/3 tablespoon

Anxiety and Confidence Questionnaires

Please complete the survey below.

Thank you!

Anxiety Questions

		Strongly disagree	Moderately disagree	Neutral	Moderately agree	Strongly agree
1)	I can be at ease and feel relaxed.					
2)	I get a frightened feeling as if something awful is about to happen.					
3)	I have worrying thoughts/anxiety about my sun exposure and sun protection.					
4)	I feel tense.					
5)	I feel I am doing something positive for my health.					
6)	I feel a sense of accomplishment.					
7)	I feel sad.					
8)	I feel helpless.					
9)	I feel in control of my health.					
10)	I experience upsetting memories of having a sunburn.					
11)	I feel comfortable about the amount of time I spend in the sun.					

Confidence Questions

		Strongly disagree	Moderately disagree	Neutral	Moderately agree	Strongly agree
12)	I am very confident that I know how to protect my skin from the sun.					
13)	I am very confident that I have good sun protection habits.					
14)	I am very confident that I will NOT forget to reapply sunscreen.					
15)	I am very confident that in summer, I will remember to wear a hat when I go outside.					

16)

I am very confident that in summer, I will be able to find shade when I am outside.

17) I am very confident that in summer, I will use sunglasses to protect my eyes when I am outside.

18) I am very confident that in summer, I will apply sunscreen before I go outside.

19) I am very confident that in summer, I will be sure to be in the shade when I am outside.

20) I am very confident that in summer, I will wear a shirt (blouse) that covers my upper arms when I am outside.

21) I am very confident that sun protection can prevent the development of skin cancer.

22) I am very confident that sun protection of my face will prevent me from getting wrinkles.

Daily Sun Habits Survey

Please complete the survey below.

Thank you!

Did you leave the house today before 6PM?	◯ Yes No
Did you spend the majority of the day in [baseline_arm_1][city]?	◯ Yes No
What state (abbreviation) where you in for the majority of the day?	__________
What city were you in for the majority of the day?	__________

Activities

Please select the range of times that you did outdoor activities and the types of activities.

Please select ALL that apply.

	Outdoor exercise (running, biking, swimming)	Outdoor relaxation (sunbathing, picnicking, reading, gardening, etc.)	Walking (commuting, errands, etc.)
6 AM - 7:59 AM	☐	☐	☐
8 AM - 9:59 AM	☐	☐	☐
10 AM - 11:59 AM	☐	☐	☐
12 PM - 1:59 PM	☐	☐	☐
2 PM - 3:59 PM	☐	☐	☐
4 PM - 6PM	☐	☐	☐

How long were you walking outside for transportation?
- ◯ 0-15 minutes
- ◯ 16-30 minutes
- ◯ 31-45 minutes
- ◯ 46 minutes to 1 hour
- ◯ Greater than 1 hour

How long were you walking outside for leisure between 6 AM to 6 PM
- ◯ 0-15 minutes
- ◯ 16-30 minutes
- ◯ 31-45 minutes
- ◯ 46 minutes to 1 hour
- ◯ Greater than 1 hour

Please select the range of times that you sweat or got wet

	Yes, I was sweating and/or got wet	No, I did not sweat and/or getwet
6 AM - 7:59 AM	◯	◯
8 AM - 9:59 AM	◯	◯
10 AM - 11:59 AM	◯	◯
12 PM - 1:59 PM	◯	◯
2 PM - 3:59 PM	◯	◯
4 PM - 6 PM	◯	◯

Please select the range of times of day that you wore sunglasses/gloves.

Select ALL that apply

	Sunglasses	Gloves
6 AM - 7:59 AM	☐	☐
8 AM - 9:59 AM	☐	☐
10 AM - 11:59 AM	☐	☐
12 PM - 1:59 PM	☐	☐
2 PM - 3:59 PM	☐	☐
4 PM - 6 PM	☐	☐

Clothing

When did you first leave the house today? ◯ AM PM

At what time did you first leave the house today? (HH: MM)

____
(For example, if you left the house at 9:05 AM, then enter 9:05. If you left the house at 1:30 PM, then enter 1:30.)

What were you wearing when you first left the house today?

Please select the options below that best match what you were wearing

Head, Neck, and Ear Coverage

Coverage Option	Coverage Example Picture	Sun Protection Provided	Sun Protection Not Provided
**1. No Coverage**	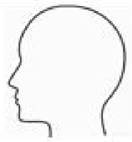	N/A	No sun protection of scalp, face, ears or neck.
**2. Visor**	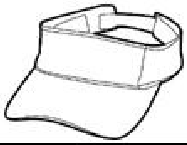	Sun protection of upper 1/3 of face	No sun protection of scalp, lower 2/3 of face, ears, or neck
**3. Baseball cap/safety hat**	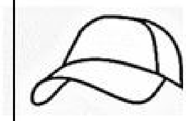 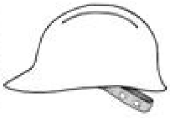	Sun protection of scalp and upper 1/3 of face	No sun protection of lower 2/3 of face, ears, and neck (back and front)
**4. 4 inch brim hat/bucket hat/sun hat**	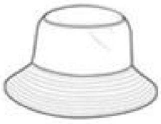 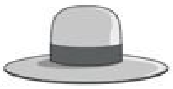	Sun protection of scalp, most of face, ears, and partial sun protection of neck	No sun protection of front of neck
**5. Sun protection hat with neck flap**	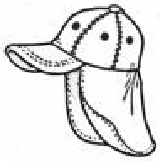	Sun protection of scalp, upper 1/3 of face, ears, and back of neck	No sun protection for lower half of face and front of neck

Please select your head, neck, and ear coverage

- ◯ 1. No coverage, bare/fully exposed head
- ◯ 2. Visor
- ◯ 3. Baseball cap/safety hat
- ◯ 4. 4-inch brim/bucket/sun hat
- ◯ 5. Sun protection hat with a neck flap

Chest, Stomach, Back, and Arm Coverage

Coverage Option	Coverage Example Picture	Sun Protection Provided	Sun Protection Not Provided
**1. No Coverage/Bikini Top/Sports Bra**	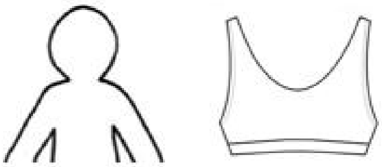	N/A	No sun protection of chest, back, shoulder or arms
**2. Tank Top**	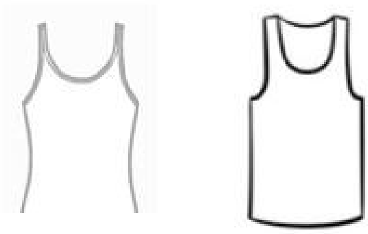	Sun protection of chest, stomach, and back	No sun protection of arms or shoulders
**3. T-Shirt**	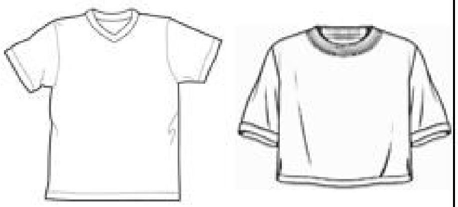	Sun protection of chest, stomach, shoulders, back and upper 1/3 of arms.	No sun protection of lower 2/3 of arms
**4. Long Sleeve**	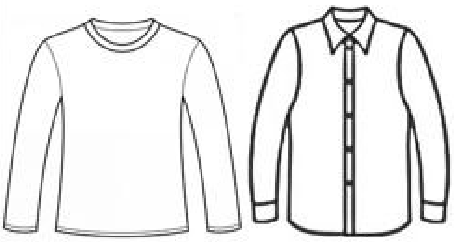	Sun protection of chest, stomach, back, and entire length of arms	N/A

Please select your chest, stomach, back, and arm coverage

- ◯ 1. No coverage/bikini top/sports bra
- ◯ 2. Tanktop
- ◯ 3. T-Shirt
- ◯ 4. Long sleeve

Leg Coverage

Coverage Option	Coverage Example Picture	Sun Protection Provided	Sun Protection Not Provided
**1. Bare Legs (bikini bottom, speedo, short shorts, mini skirt)**	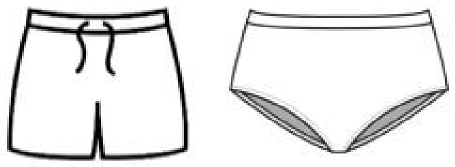	N/A	No sun protection of legs or ankles
**2. Knee-length shorts or skirt**	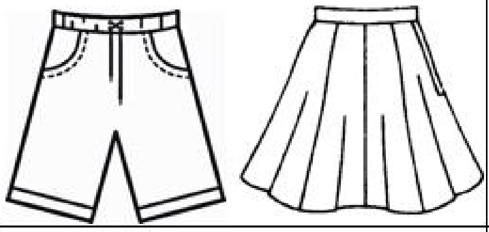	Sun protection of upper ½ of legs.	No sun protection of lower 1/2 of legs or ankles
**3. Capri (mid-calf) length pants/skirts**	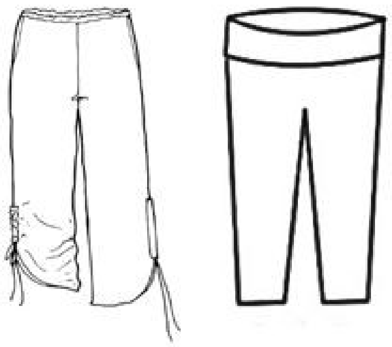	Sun protection of 3/4th of legs	No sun protection of lower ¼ of leg ankles
**4. Long pants or skirt**	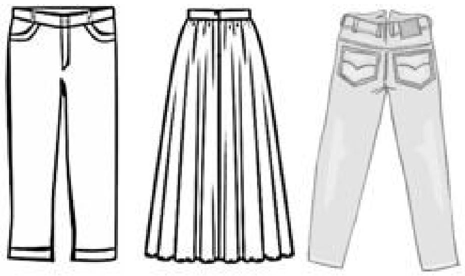	Sun protection of legs and ankles	N/A

Please select your leg coverage

- ◯ 1. Bare legs/short shorts/bikini bottom/speedo
- ◯ 2. Knee-length shorts or skirt
- ◯ 3. Mid-calf length shorts/skirts/capris
- ◯ 4. Long pants

Foot Coverage

Coverage Option	Coverage Example Picture	Sun Protection Provided	Sun Protection Not Provided
1. Bare feet/flip flops/sandals	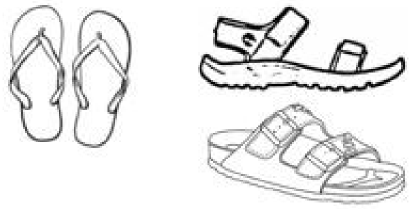	N/A	No/very minimal sun protection to foot
2. Sneaker/Closed Toe shoe	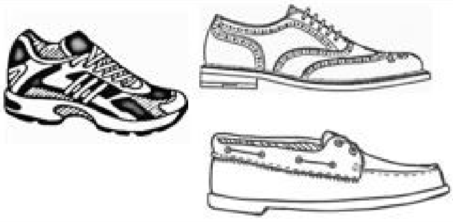	Sun protection of entire foot	No sun protection of ankle
3. Ankle Shoes/Boots	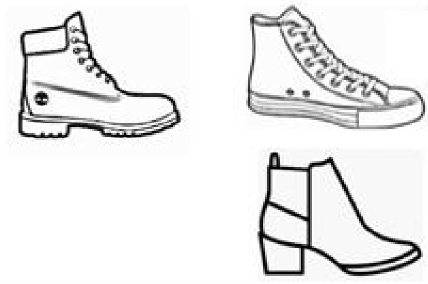	Sun protection of entire foot and ankle	No sun protection
4. Calf high shoes/boots	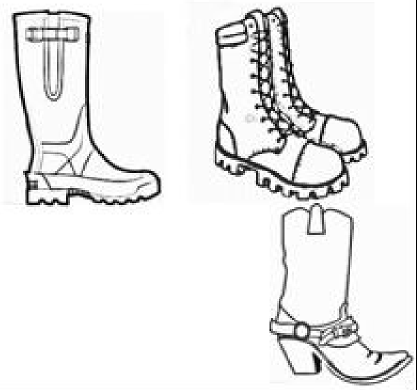	Sun protection of entire foot, ankle, bottom third of legs	N/A

Please select your foot coverage

- ☐ 1. Bare feet/flip flops/sandals
- ☐ 2. Sneakers/closed-toe shoes
- ☐ 3. Ankle shoes/boots
- ☐ 4. Calf-high shoes/boots

Did any of your clothing have Ultraviolet Protection Factor (UPF) of 50 or more?

- ◯ Yes
- ◯ No
- ◯ Don't know

What body areas were covered by clothing with a UPF of 50 or more?<br><br>(select all that apply)	☐ Scalp<br>☐ Face<br>☐ Ears<br>☐ Neck<br>☐ Shoulders<br>☐ Back<br>☐ Chest<br>☐ Stomach<br>☐ Arms<br>☐ Hands<br>☐ Buttocks<br>☐ Legs<br>☐ Feet
Did you change clothes later in the day (including removing or adding any layers)?	◯ Yes ◯ No
When did you first add or remove clothing?	◯ AM ◯ PM
Stop! Clothing times not in order! First time was in the "PM"	
At what time did you first add or remove clothing (HH: MM)	____________________<br>(For example, if changed clothing at 10:05 AM, then enter 10:05. If you changed clothes at 2:30 PM, then enter 2:30.)
Stop! Clothing times not in order! First time was later than second time	
Please select the clothing you were wearing when you added or removed clothing for the first time	

Head, Neck, and Ear Coverage

Coverage Option	Coverage Example Picture	Sun Protection Provided	Sun Protection Not Provided
**1. No Coverage**	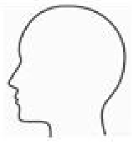	N/A	No sun protection of scalp, face, ears or neck.
**2. Visor**	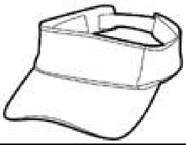	Sun protection of upper 1/3 of face	No sun protection of scalp, lower 2/3 of face, ears, or neck
**3. Baseball cap/safety hat**	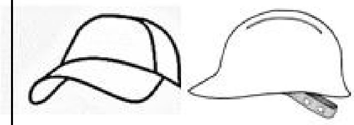	Sun protection of scalp and upper 1/3 of face	No sun protection of lower 2/3 of face, ears, and neck (back and front)
**4. 4 inch brim hat/bucket hat/sun hat**	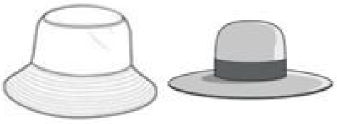	Sun protection of scalp, most of face, ears, and partial sun protection of neck	No sun protection of front of neck
**5. Sun protection hat with neck flap**	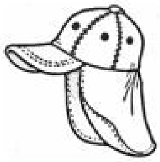	Sun protection of scalp, upper 1/3 of face, ears, and back of neck	No sun protection for lower half of face and front of neck

Please select your head, neck, and ear coverage

- ◯ 1. No coverage, bare/fully exposed head
- ◯ 2. Visor
- ◯ 3. Baseball cap/safety hat
- ◯ 4. 4-inch brim/bucket/sun hat
- ◯ 5. Sun protection hat with a neck flap

Chest, Stomach, Back, and Arm Coverage

Coverage Option	Coverage Example Picture	Sun Protection Provided	Sun Protection Not Provided
1. No Coverage/Bikini Top/Sports Bra	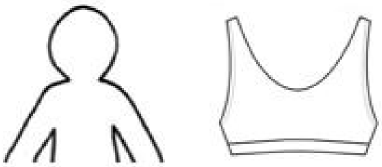	N/A	No sun protection of chest, back, shoulder or arms
2. Tank Top	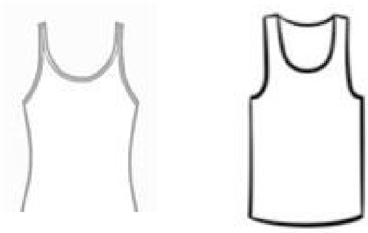	Sun protection of chest, stomach, and back	No sun protection of arms or shoulders
3. T-Shirt	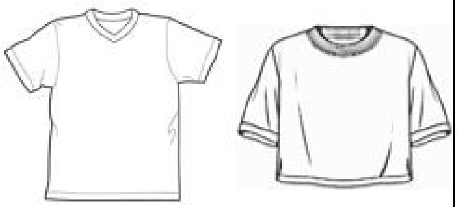	Sun protection of chest, stomach, shoulders, back and upper 1/3 of arms.	No sun protection of lower 2/3 of arms
4. Long Sleeve	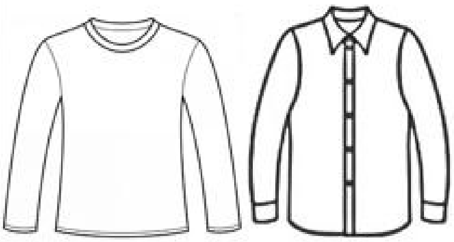	Sun protection of chest, stomach, back, and entire length of arms	N/A

Please select your chest, stomach, back, and arm coverage

- ◯ 1. No coverage/bikini top/sports bra
- ◯ 2. Tanktop
- ◯ 3. T-Shirt
- ◯ 4. Long sleeve

Leg Coverage

Coverage Option	Coverage Example Picture	Sun Protection Provided	Sun Protection Not Provided
**1. Bare Legs (bikini bottom, speedo, short shorts, mini skirt)**	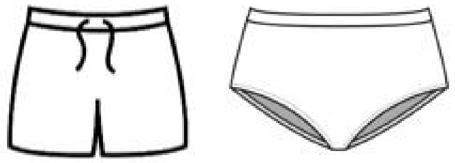	N/A	No sun protection of legs or ankles
**2. Knee-length shorts or skirt**	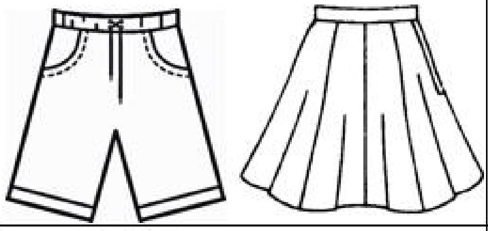	Sun protection of upper ½ of legs.	No sun protection of lower 1/2 of legs or ankles
**3. Capri (mid-calf) length pants/skirts**	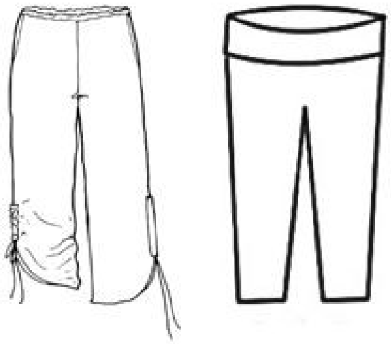	Sun protection of $3/4^{th}$ of legs	No sun protection of lower ¼ of leg ankles
**4. Long pants or skirt**	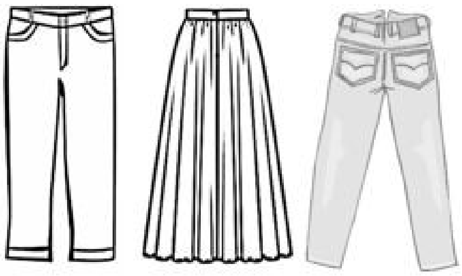	Sun protection of legs and ankles	N/A

Please select your leg coverage

◯ 1. Bare legs/short shorts/bikini bottom/speedo
◯ 2. Knee-length shorts or skirt
◯ 3. Mid-calf length shorts/skirts/capris
◯ 4. Long pants

Foot Coverage

Coverage Option	Coverage Example Picture	Sun Protection Provided	Sun Protection Not Provided
1. Bare feet/flip flops/sandals	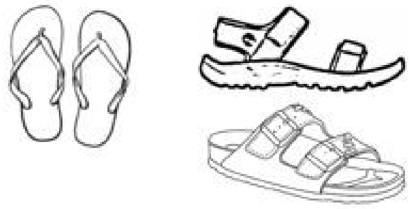	N/A	No/very minimal sun protection to foot
2. Sneaker/Closed Toe shoe	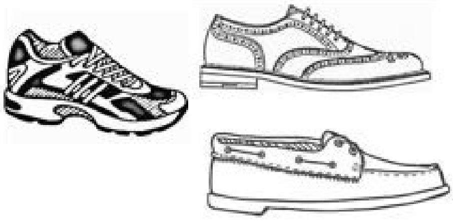	Sun protection of entire foot	No sun protection of ankle
3. Ankle Shoes/Boots	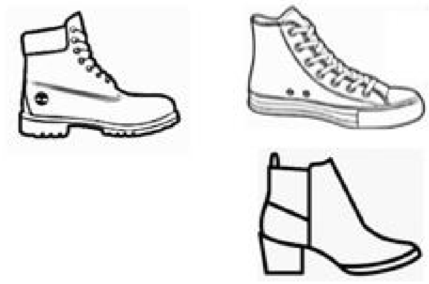	Sun protection of entire foot and ankle	No sun protection
4. Calf high shoes/boots	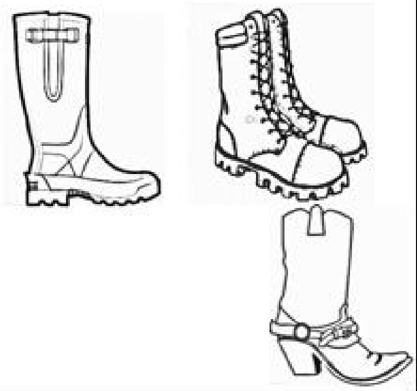	Sun protection of entire foot, ankle, bottom third of legs	N/A

Please select your foot coverage

◯ 1. Bare feet/flip flops/sandals
◯ 2. Sneakers/closed-toe shoes
◯ 3. Ankle shoes/boots
◯ 4. Calf-high shoes/boots

Did you add or remove clothing a second time? ◯ Yes ◯ No

When did you add or remove clothing for the second time? ◯ AM ◯ PM

Stop! Clothing times not in order! Second time was in the "PM"

What time did you add or remove for the second time? (HH: MM)

____
(For example, if changed clothing at 11:05 AM, then enter 11:05. If you changed clothes at 3:30 PM, then enter 3:30.)

Stop! Clothing times not in order! Second time was later than third time

Please select what you were wearing when you changed clothes (added or removed) for the second time

Head, Neck, and Ear Coverage

Coverage Option	Coverage Example Picture	Sun Protection Provided	Sun Protection Not Provided
**1. No Coverage**	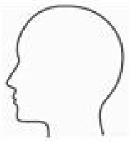	N/A	No sun protection of scalp, face, ears or neck.
**2. Visor**	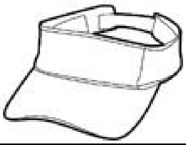	Sun protection of upper 1/3 of face	No sun protection of scalp, lower 2/3 of face, ears, or neck
**3. Baseball cap/safety hat**	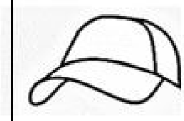 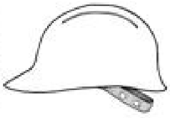	Sun protection of scalp and upper 1/3 of face	No sun protection of lower 2/3 of face, ears, and neck (back and front)
**4. 4 inch brim hat/bucket hat/sun hat**	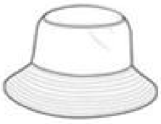 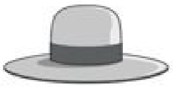	Sun protection of scalp, most of face, ears, and partial sun protection of neck	No sun protection of front of neck
**5. Sun protection hat with neck flap**	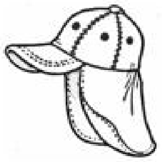	Sun protection of scalp, upper 1/3 of face, ears, and back of neck	No sun protection for lower half of face and front of neck

Please select your head, neck, and ear coverage

- ◯ 1. No coverage, bare/fully exposed head
- ◯ 2. Visor
- ◯ 3. Baseball cap/safety hat
- ◯ 4. 4-inch brim/bucket/sun hat
- ◯ 5. Sun protection hat with a neck flap

Chest, Stomach, Back, and Arm Coverage

Coverage Option	Coverage Example Picture	Sun Protection Provided	Sun Protection Not Provided
**1. No Coverage/Bikini Top/Sports Bra**	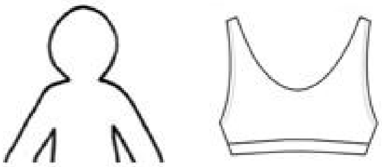	N/A	No sun protection of chest, back, shoulder or arms
**2. Tank Top**	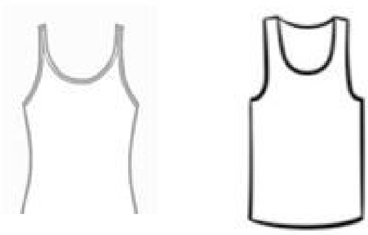	Sun protection of chest, stomach, and back	No sun protection of arms or shoulders
**3. T-Shirt**	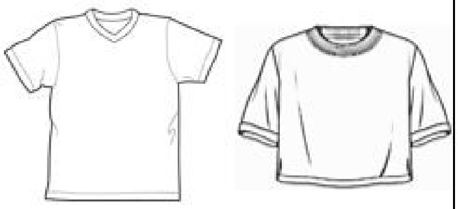	Sun protection of chest, stomach, shoulders, back and upper 1/3 of arms.	No sun protection of lower 2/3 of arms
**4. Long Sleeve**	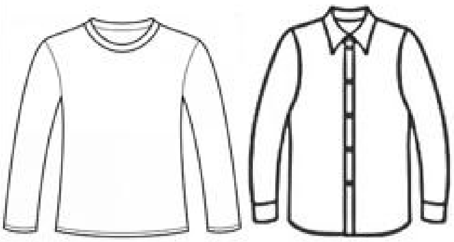	Sun protection of chest, stomach, back, and entire length of arms	N/A

Please select your chest, stomach, back, and arm coverage

- ◯ 1. No coverage/bikini top/sports bra
- ◯ 2. Tanktop
- ◯ 3. T-Shirt
- ◯ 4. Long sleeve

Leg Coverage

Coverage Option	Coverage Example Picture	Sun Protection Provided	Sun Protection Not Provided
**1. Bare Legs (bikini bottom, speedo, short shorts, mini skirt)**	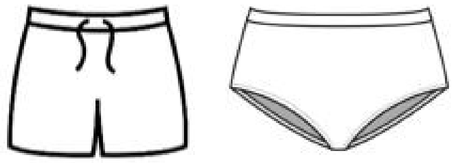	N/A	No sun protection of legs or ankles
**2. Knee-length shorts or skirt**	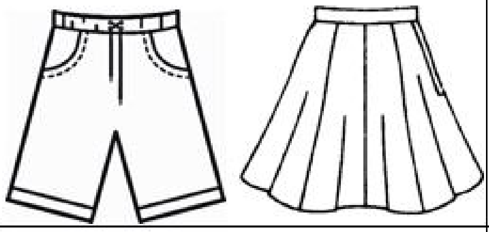	Sun protection of upper ½ of legs.	No sun protection of lower 1/2 of legs or ankles
**3. Capri (mid-calf) length pants/skirts**	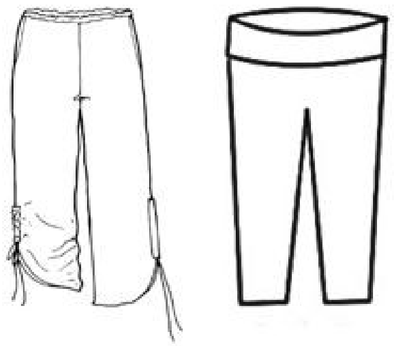	Sun protection of 3/4th of legs	No sun protection of lower ¼ of leg ankles
**4. Long pants or skirt**	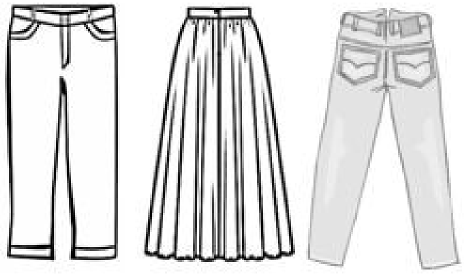	Sun protection of legs and ankles	N/A

Please select your leg coverage

◯ 1. Bare legs/short shorts/bikini bottom/speedo
◯ 2. Knee-length shorts or skirt
◯ 3. Mid-calf length shorts/skirts/capris
◯ 4. Long pants

Foot Coverage

Please select your foot coverage

◯ 1. Bare feet/flip flops/sandals
◯ 2. Sneakers/closed-toe shoes
◯ 3. Ankle shoes/boots
◯ 4. Calf-high shoes/boots

Did you add or remove clothing a third time?	◯ Yes ◯ No
When did you add or remove clothing for the third time?	◯ AM PM
Stop! Clothing times not in order! Third time was in the "PM"	
What time did you add or remove for the third time? (HH: MM)	____________________ (For example, if changed clothing at noon, then enter 12:00. If you changed clothes at 4:30 PM, then enter 4:30.)
Stop! Clothing times not in order! Third time was later than fourth time	

Please select what you were wearing when you changed clothes (added or removed) for the third time

Head, Neck, and Ear Coverage

**Coverage Option**	**Coverage Example Picture**	**Sun Protection Provided**	**Sun Protection Not Provided**
**1. No Coverage**	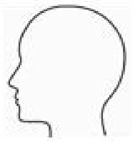	N/A	No sun protection of scalp, face, ears or neck.
**2. Visor**	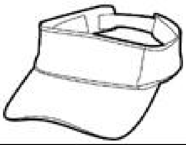	Sun protection of upper 1/3 of face	No sun protection of scalp, lower 2/3 of face, ears, or neck
**3. Baseball cap/safety hat**	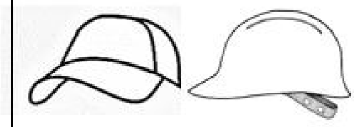	Sun protection of scalp and upper 1/3 of face	No sun protection of lower 2/3 of face, ears, and neck (back and front)
**4. 4 inch brim hat/bucket hat/sun hat**	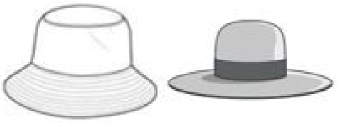	Sun protection of scalp, most of face, ears, and partial sun protection of neck	No sun protection of front of neck
**5. Sun protection hat with neck flap**	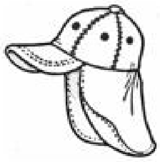	Sun protection of scalp, upper 1/3 of face, ears, and back of neck	No sun protection for lower half of face and front of neck

Please select your head, neck, and ear coverage	◯ 1. No coverage, bare/fully exposed head<br>◯ 2. Visor<br>◯ 3. Baseball cap/safety hat<br>◯ 4. 4-inch brim/bucket/sun hat<br>◯ 5. Sun protection hat with a neck flap

Chest, Stomach, Back, and Arm Coverage

Coverage Option	Coverage Example Picture	Sun Protection Provided	Sun Protection Not Provided
**1. No Coverage/Bikini Top/Sports Bra**	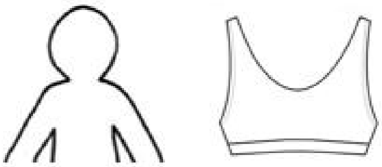	N/A	No sun protection of chest, back, shoulder or arms
**2. Tank Top**	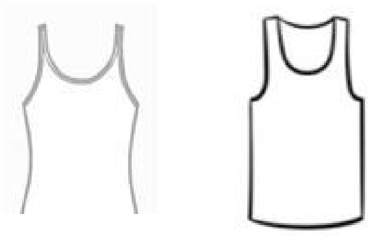	Sun protection of chest, stomach, and back	No sun protection of arms or shoulders
**3. T-Shirt**	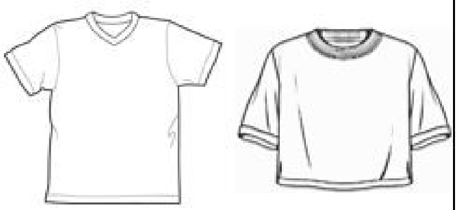	Sun protection of chest, stomach, shoulders, back and upper 1/3 of arms.	No sun protection of lower 2/3 of arms
**4. Long Sleeve**	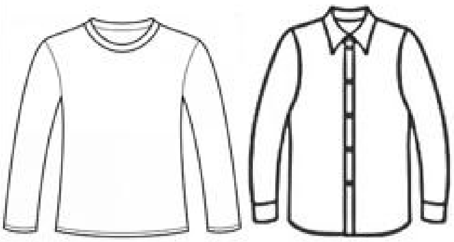	Sun protection of chest, stomach, back, and entire length of arms	N/A

Please select your chest, stomach, back, and arm coverage

◯ 1. No coverage/bikini top/sports bra
◯ 2. Tanktop
◯ 3. T-Shirt
◯ 4. Long sleeve

Leg Coverage

Coverage Option	Coverage Example Picture	Sun Protection Provided	Sun Protection Not Provided
**1. Bare Legs (bikini bottom, speedo, short shorts, mini skirt)**	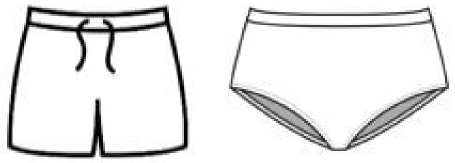	N/A	No sun protection of legs or ankles
**2. Knee-length shorts or skirt**	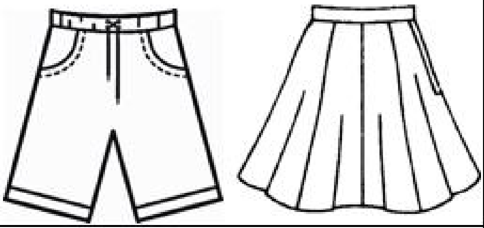	Sun protection of upper ½ of legs.	No sun protection of lower 1/2 of legs or ankles
**3. Capri (mid-calf) length pants/skirts**	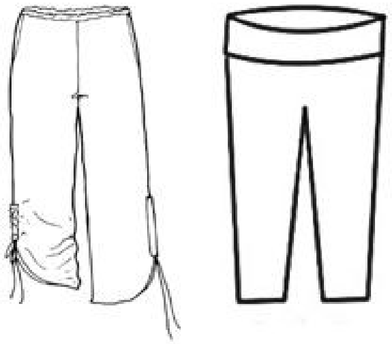	Sun protection of 3/4th of legs	No sun protection of lower ¼ of leg ankles
**4. Long pants or skirt**	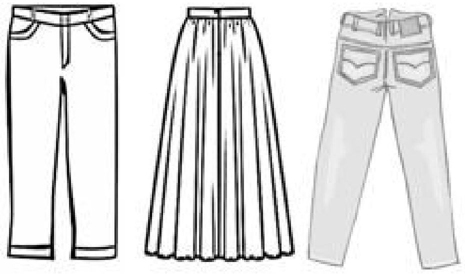	Sun protection of legs and ankles	N/A

Please select your leg coverage

◯ 1. Bare legs/short shorts/bikini bottom/speedo
◯ 2. Knee-length shorts or skirt
◯ 3. Mid-calf length shorts/skirts/capris
◯ 4. Long pants

Foot Coverage

Coverage Option	Coverage Example Picture	Sun Protection Provided	Sun Protection Not Provided
1. Bare feet/flip flops/sandals	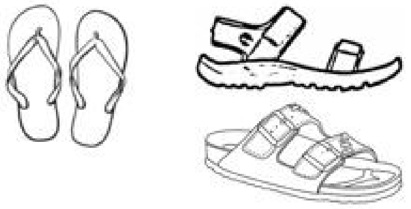	N/A	No/very minimal sun protection to foot
2. Sneaker/Closed Toe shoe	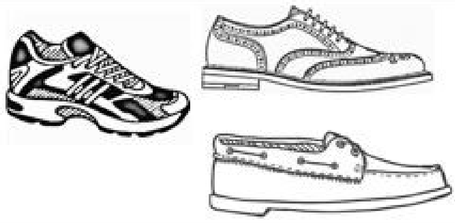	Sun protection of entire foot	No sun protection of ankle
3. Ankle Shoes/Boots	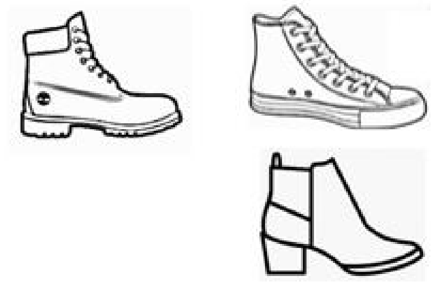	Sun protection of entire foot and ankle	No sun protection
4. Calf high shoes/boots	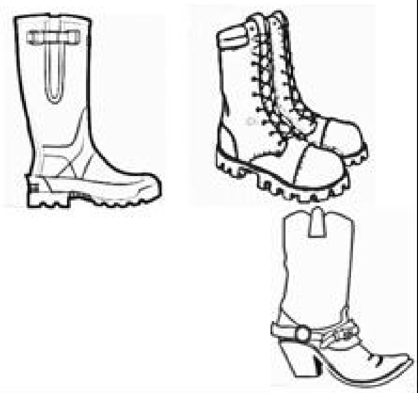	Sun protection of entire foot, ankle, bottom third of legs	N/A

Please select your foot coverage

◯ 1. Bare feet/flip flops/sandals
◯ 2. Sneakers/closed-toe shoes
◯ 3. Ankle shoes/boots
◯ 4. Calf-high shoes/boots

Sunscreen

Did you put on sunscreen today? ◯ Yes ◯ No

When did you first apply sunscreen? (AM or PM) ◯ AM ◯ PM

At what time did you first apply sunscreen? (HH:MM)

____
(For example, if you put sunscreen on at 10:05 AM, then enter 10:05. If you put sunscreen on at 2:30 PM, then enter 2:30.)

When you first applied sunscreen, how many types of sunscreen did you use?	◯ One<br>◯ Two<br>◯ Three (or more)
Please select the SPF of the first type of sunscreen you applied	◯ SPF 0-14<br>◯ SPF 15-49<br>◯ SPF 50 or greater
Which part (s) of your body did you apply your first type of sunscreen to?<br>(select all that apply)	☐ Scalp<br>☐ Face<br>☐ Ears<br>☐ Neck<br>☐ Shoulders<br>☐ Back<br>☐ Chest<br>☐ Stomach<br>☐ Arms<br>☐ Hands<br>☐ Buttocks<br>☐ Legs<br>☐ Feet
Please select the SPF of the second type of sunscreen you applied	◯ SPF 0-14<br>◯ SPF 15-49<br>◯ SPF 50 or greater
Which part (s) of your body did you apply your second type of sunscreen to? (select all that apply)	☐ Scalp<br>☐ Face<br>☐ Ears<br>☐ Neck<br>☐ Shoulders<br>☐ Back<br>☐ Chest<br>☐ Stomach<br>☐ Arms<br>☐ Hands<br>☐ Buttocks<br>☐ Legs<br>☐ Feet
Please select the SPF of the third type of sunscreen you applied	◯ SPF 0-14<br>◯ SPF 15-49<br>◯ SPF 50 or greater
Which part (s) of your body did you apply your third type of sunscreen to? (select all that apply)	☐ Scalp<br>☐ Face<br>☐ Ears<br>☐ Neck<br>☐ Shoulders<br>☐ Back<br>☐ Chest<br>☐ Stomach<br>☐ Arms<br>☐ Hands<br>☐ Buttocks<br>☐ Legs<br>☐ Feet
Did you reapply sunscreen at any time today?	◯ Yes ◯ No

How many times did you reapply sunscreen today?	◯ Once<br>◯ Twice<br>◯ Three times (or more) times
When did you reapply sunscreen for the first time? (AM or PM)	◯ AM ◯ PM
Stop! Sunscreen times not in order! First time was in the "PM"	
At what time did you reapply sunscreen for the first time? (HH: MM)	__________________________<br>(For example, if you reapplied sunscreen at 11:15 AM, then enter 11:15. If you reapplied sunscreen at 3:30 PM, then enter 3:30.)
Stop! Sunscreen times not in order! First time was later than second time	
When you reapplied sunscreen for the first time, how many types of sunscreen didyou use?	◯ One<br>◯ Two<br>◯ Three (or more)
Please select the SPF of the first type of sunscreen you applied	◯ SPF 0-14<br>◯ SPF 15-49<br>◯ SPF 50 or greater
Which part (s) of your body did you apply your first type of sunscreen to? (select all that apply)	☐ Scalp<br>☐ Face<br>☐ Ears<br>☐ Neck<br>☐ Shoulders<br>☐ Back<br>☐ Chest<br>☐ Stomach<br>☐ Arms<br>☐ Hands<br>☐ Buttocks<br>☐ Legs<br>☐ Feet
Please select the SPF of the second type of sunscreen you applied	◯ SPF 0-14<br>◯ SPF 15-49<br>◯ SPF 50 or greater
Which part (s) of your body did you apply your second type of sunscreen to? (select all that apply)	☐ Scalp<br>☐ Face<br>☐ Ears<br>☐ Neck<br>☐ Shoulders<br>☐ Back<br>☐ Chest<br>☐ Stomach<br>☐ Arms<br>☐ Hands<br>☐ Buttocks<br>☐ Legs<br>☐ Feet

Please select the SPF of the third type of sunscreen you applied	◯ SPF 0-14<br>◯ SPF 15-49<br>◯ SPF 50 or greater
Which part (s) of your body did you apply your third type of sunscreen to? (select all that apply)	☐ Scalp<br>☐ Face<br>☐ Ears<br>☐ Neck<br>☐ Shoulders<br>☐ Back<br>☐ Chest<br>☐ Stomach<br>☐ Arms<br>☐ Hands<br>☐ Buttocks<br>☐ Legs<br>☐ Feet
When did you reapply sunscreen for the second time? (AM or PM)	◯ AM ◯ PM
Stop! Sunscreen times not in order! Second time was in the "PM"	
At what time did you reapply sunscreen for the second time? (HH: MM) For example, if you reapplied sunscreen a second time at 11:45 AM, then enter 11:45. If you reapplied sunscreen a second time at 4:10 PM, then enter 4:10.	______________________
Stop! Sunscreen times not in order! Second time was later than third time	
When you reapplied sunscreen for the second time, how many types of sunscreen did you use?	◯ One<br>◯ Two<br>◯ Three (or more)
Please select the SPF of the first type of sunscreen you applied	◯ SPF 0-14<br>◯ SPF 15-49<br>◯ SPF 50 or greater
Which part (s) of your body did you apply your first type of sunscreen to? (select all that apply)	☐ Scalp<br>☐ Face<br>☐ Ears<br>☐ Neck<br>☐ Shoulders<br>☐ Back<br>☐ Chest<br>☐ Stomach<br>☐ Arms<br>☐ Hands<br>☐ Buttocks<br>☐ Legs<br>☐ Feet
Please select the SPF of the second type of sunscreen you applied	◯ SPF 0-14<br>◯ SPF 15-49<br>◯ SPF 50 or greater

Which part (s) of your body did you apply your second type of sunscreen to? (select all that apply)	☐ Scalp<br>☐ Face<br>☐ Ears<br>☐ Neck<br>☐ Shoulders<br>☐ Back<br>☐ Chest<br>☐ Stomach<br>☐ Arms<br>☐ Hands<br>☐ Buttocks<br>☐ Legs<br>☐ Feet
Please select the SPF of the third type of sunscreen you applied	◯ SPF 0-14<br>◯ SPF 15-49<br>◯ SPF 50 or greater
Which part (s) of your body did you apply your third type of sunscreen to? (select all that apply)	☐ Scalp<br>☐ Face<br>☐ Ears<br>☐ Neck<br>☐ Shoulders<br>☐ Back<br>☐ Chest<br>☐ Stomach<br>☐ Arms<br>☐ Hands<br>☐ Buttocks<br>☐ Legs<br>☐ Feet
When did you reapply sunscreen for the third time? (AM or PM)	◯ AM ◯ PM
Stop! Sunscreen times not in order! Third time was in the "PM"	
At what time did you reapply sunscreen for the third time? (HH: MM)	____________________<br>(For example, if you reapplied sunscreen at noon, then enter 12:00. If you reapplied sunscreen at 3:35 PM, then enter 3:35.)
Stop! Sunscreen times not in order! Third time was later than fourth time	
When you reapplied sunscreen for the third time, how many types of sunscreen did you use?	◯ One<br>◯ Two<br>◯ Three (or more)
Please select the SPF of the first type of sunscreen you applied	◯ SPF 0-14<br>◯ SPF 15-49<br>◯ SPF 50 or greater

Which part (s) of your body did you apply your first type of sunscreen to? (select all that apply)	☐ Scalp<br>☐ Face<br>☐ Ears<br>☐ Neck<br>☐ Shoulders<br>☐ Back<br>☐ Chest<br>☐ Stomach<br>☐ Arms<br>☐ Hands<br>☐ Buttocks<br>☐ Legs<br>☐ Feet
Please select the SPF of the second type of sunscreen you applied	◯ SPF 0-14<br>◯ SPF 15-49<br>◯ SPF 50 or greater
Which part (s) of your body did you apply your second type of sunscreen to? (select all that apply)	☐ Scalp<br>☐ Face<br>☐ Ears<br>☐ Neck<br>☐ Shoulders<br>☐ Back<br>☐ Chest<br>☐ Stomach<br>☐ Arms<br>☐ Hands<br>☐ Buttocks<br>☐ Legs<br>☐ Feet
Please select the SPF of the third type of sunscreen you applied	◯ SPF 0-14<br>◯ SPF 15-49<br>◯ SPF 50 or greater
Which part (s) of your body did you apply your third type of sunscreen to? (select all that apply)	☐ Scalp<br>☐ Face<br>☐ Ears<br>☐ Neck<br>☐ Shoulders<br>☐ Back<br>☐ Chest<br>☐ Stomach<br>☐ Arms<br>☐ Hands<br>☐ Buttocks<br>☐ Legs<br>☐ Feet

Sunburn

Did you sunburn on even a small part of your skin, today?

◯ Yes ◯ No

Which part(s) of your body got a sunburn today: (select all that apply)

- ☐ Scalp
- ☐ Face
- ☐ Ears
- ☐ Neck
- ☐ Shoulders
- ☐ Back
- ☐ Chest
- ☐ Stomach
- ☐ Arms
- ☐ Hands
- ☐ Buttocks
- ☐ Legs
- ☐ Feet

Goal Survey

Please complete the survey below.

Thank you!

How will you meet your goal to engage in sun-protected outdoor activity? (Select up to TWO)<br><br>Tomorrow, I will	☐ Apply sunscreen to all of the areas of my body that may be exposed to the sun<br>☐ Apply sunscreen before I go outdoors<br>☐ Wear a hat when I am outdoors<br>☐ Wear a shirt that covers my shoulders when I am outdoors<br>☐ Plan my outdoor activities to avoid being outside from 10 AM to 2 PM<br>☐ Pay attention to the strength of the sun by checking the UV Guard report 15 minutes after I go outside<br>☐ Be careful not to exceed the amount of UV my skin can tolerate

Goal Followup Survey

Please complete the survey below.

Thank you!

Yesterday you picked as a goal:<br><br>"Apply sunscreen to all of the areas of my body that may be exposed to the sun"<br><br>Do you intend to keep doing this?	◯ Yes No
Yesterday you picked as a goal:<br><br>"Apply sunscreen before I go outdoors"<br><br>Do you intend to keep doing this?	◯ Yes No
Yesterday you picked as a goal:<br><br>"Wear a hat when I am outdoors"<br><br>Do you intend to keep doing this?	◯ Yes No
Yesterday you picked as a goal:<br><br>"Wear a shirt that covers my shoulders when I am outdoors"<br><br>Do you intend to keep doing this?	◯ Yes No
Yesterday you picked as a goal:<br><br>"Plan my outdoor activities to avoid being outside from 10 AM to 2 PM"<br><br>Do you intend to keep doing this?	◯ Yes No
Yesterday you picked as a goal:<br><br>"Pay attention to the strength of the sun by checking the UV Guard report 15 minutes after I go outside"<br><br>Do you intend to keep doing this?	◯ Yes No
Yesterday you picked as a goal:<br><br>"Be careful not to exceed the amount of UV my skin can tolerate"<br><br>Do you intend to keep doing this?	◯ Yes No

System Usability Scale

Please complete the survey below.

Thank you!

		Strongly Disagree	Disagree	Neither Agree nor Disagree	Agree	Strongly Agree
1)	1. I think that I would like to use this system frequently.					
2)	2. I found the system unnecessarily complex.					
3)	3. I thought the system was easy to use.					
4)	4. I think that I would need the support of a technical person to be able to use this system.					
5)	5. I found the various functions in this system were well integrated.					
6)	6. I thought there was too much inconsistency in this system.					
7)	7. I would imagine that most people would learn to use this system very quickly.					
8)	8. I found the system very cumbersome to use.					
9)	9. I felt very confident using the system.					
10)	10. I needed to learn a lot of things before I could get going with this system.					

Exit Questions

Please complete the survey below.

Thank you!

Compared to last summer, do you feel that you spent LESS time outdoors this summer?	◯ Yes, a lot<br>◯ Yes, a little<br>◯ About the same<br>◯ No, a little more<br>◯ No, a lot more
Why do you think the time you spent outdoors this summer was different from last year? (select as many as apply)	☐ COVID-19<br>☐ Weather<br>☐ Work<br>☐ Sports<br>☐ Other
Other:	__________________________________
Were there instances where you went outside while wearing the UV sensor without your phone nearby (within 2 feet)?	◯ Yes, often<br>◯ Yes, sometimes<br>◯ No
How did you feel about wearing the UV sensor?	◯ Liked<br>◯ Neither liked nor disliked<br>◯ Disliked
Were there any days that you did not to wear the UV?	◯ Yes, often<br>◯ Yes, sometimes<br>◯ No, I wore it every day
Why did you choose not to wear the UV sensor? (select as many as apply)	☐ Forgot<br>☐ Appearance of sensor<br>☐ Weather<br>☐ Sweat under sensor<br>☐ Tan lines created by wearing sensor on wrist<br>☐ Other
Other:	__________________________________
How willing are you to continue using the UV sensor and the UV Guard app as part of research?	◯ Extremely willing<br>◯ Moderately willing<br>◯ Somewhat willing<br>◯ Slightly willing<br>◯ Not willing
How willing are you to continue using the UV sensor and the UV Guard app personally?	◯ Extremely willing<br>◯ Moderately willing<br>◯ Somewhat willing<br>◯ Slightly willing<br>◯ Not willing

How often would you use the UV sensor and the UV Guard app?	◯ Every day<br>◯ Few times per week<br>◯ Few times per month
Are there any features you would like to see added to the UV sensor or the UV Guard app?	__________________________
How often did you open the UV Guard to track your UV exposure?	◯ Multiple times per day<br>◯ Once per day<br>◯ Once every few days<br>◯ Rarely
Did you change the times you went outside based on your observed UV exposure?	◯ Yes ◯ No
Did you shift the time you went outside to periods with less intense UV exposure? For example, did you go outside early in the morning or late in the afternoon?	◯ Yes ◯ No
Did you change how much time you spent outside based on your observed UV exposure as seen on the graph presented on your phone?	◯ Yes ◯ No
Did you change your use of sun protection based on your observed UV exposure as seen on the graph presented on your phone?	◯ Yes ◯ No
How did you change your use of sun protection?	__________________________
